# Diagnostic Accuracy of Rapid Antigen Test Kits for Detecting SARS-CoV-2: A Systematic Review and Meta-Analysis of 17,171 Suspected COVID-19 Patients

**DOI:** 10.3390/jcm10163493

**Published:** 2021-08-08

**Authors:** Shahad Saif Khandker, Nik Haszroel Hysham Nik Hashim, Zakuan Zainy Deris, Rafidah Hanim Shueb, Md Asiful Islam

**Affiliations:** 1 Gonoshasthaya-RNA Molecular Diagnostic and Research Center, Dhanmondi, Dhaka 1205, Bangladesh; shahad@rnabiotech.com.bd; 2 Department of Medical Microbiology and Parasitology, School of Medical Sciences, Universiti Sains Malaysia, Kubang Kerian 16150, Kelantan, Malaysia; haszroel@usm.my (N.H.H.N.H.); zakuan@usm.my (Z.Z.D.); 3 Medical Microbiology Laboratory, Hospital Universiti Sains Malaysia, Kubang Kerian 16150, Kelantan, Malaysia; 4 Department of Haematology, School of Medical Sciences, Universiti Sains Malaysia, Kubang Kerian 16150, Kelantan, Malaysia

**Keywords:** COVID-19, SARS-CoV-2, rapid antigen test, specificity, sensitivity

## Abstract

Early diagnosis is still as crucial as the initial stage of the COVID-19 pandemic. As RT-PCR sometimes is not feasible in developing nations or rural areas, health professionals may use a rapid antigen test (RAT) to lessen the load of diagnosis. However, the efficacy of RAT is yet to be investigated thoroughly. Hence, we tried to evaluate the overall performance of RAT in SARS-CoV-2 diagnosis. Based on our PROSPERO registered protocol (CRD42021231432), we searched online databases (i.e., PubMed, Google Scholar, Scopus, and Web of Science) and analysed overall pooled specificity and sensitivity of RAT along with study quality, publication bias, heterogeneity and more. The overall pooled specificity and sensitivity of RAT were detected as 99.4% (95% CI: 99.1–99.8; *I^2^* = 90%) and 68.4% (95% CI: 60.8–75.9; *I*^2^ = 98%), respectively. In subgroup analyses, nasopharyngeal specimens and symptomatic patient’s samples were more sensitive in RAT, while cycle threshold (Ct) values were found to have an inverse relationship with sensitivity. In the European and American populations, RAT showed better performance. Although the sensitivity of RAT is yet to be improved, it could still be an alternative in places with poor laboratory set up. Nevertheless, the negative samples of RAT can be re-tested using RT-PCR to reduce false negative results.

## 1. Introduction

Severe acute respiratory syndrome coronavirus 2 (SARS-CoV-2) is responsible for the coronavirus disease 2019 (COVID-19), characterised mainly by fever, cough, sore throat, fatigue, joint and muscle pain and loss of smell and taste [1,2,3,4,5]. It was first identified in Hubei province in Wuhan, China, in December 2019 and until now, it continues to be a significant health issue worldwide. Because there is no specific therapy or medication, early and accurate diagnosis is critical in preventing the spread of the disease [6,7]. In vitro diagnostics have long been recognised as a valuable tool for outbreak control and patient care [8]. The current gold standard diagnostic test for laboratory diagnosis of SARS-CoV-2 is nucleic acid amplification tests (NAAT); reverse transcription polymerase chain reaction (RT-PCR) to be specific [9]. The advantage this has over other tests is its high sensitivity due to the nucleic acid amplification step while also having a high specificity due to the specific primers used. This is consistent with its purpose at referral centres, which is to advise clinical care of patients. However, since it can take several hours to obtain results from RT-PCR, it may be inappropriate to use during an emergency. It also requires costly technology and competent workers, both of which may not be accessible in remote health clinics, particularly in underdeveloped countries.

Rapid antigen test (RAT) kits have emerged as an important alternative tool to aid in the clinical diagnosis of COVID-19. RAT is based on immunochromatography, which employs antibodies spotted onto nitrocellulose membranes that interact with specific antigens from the patient sample. The antigen–antibody interaction can be visualised manually or by using an immunofluorescence machine reader. For the diagnosis of COVID-19, the target analyte is often the virus’ nucleocapsid protein. A similar strategy has been used for the rapid diagnosis of HIV, malaria, influenza and other diseases [9]. It is a reasonably inexpensive and simple test with quick results that may be used for point-of-care testing [10].

The advantage of RAT is that the test is more accessible to patients. It also enables appropriate infection control measures to be instituted earlier which is important in a pandemic [10,11]. However, a negative RAT result cannot rule out COVID-19 infection [12]. Patients with typical clinical presentation or close contacts of COVID-19 cases should undergo repeat testing [12,13].

For diagnosis of SARS-CoV-2, the World Health Organisation has recommended that a RAT kit needs to meet a minimum performance requirement of at least 80% sensitivity and 97% specificity compared with a NAAT reference assay to be used [14]. Studies regarding the diagnostic accuracy of RAT have produced a wide range of sensitivity. The varied results may be due to the various study designs, manufacturer of the RAT kits, patient selection, types of specimens and the phase of illness at the point of sample collection. While research and development of RAT to detect SARS-CoV-2 continue, this systematic review and meta-analysis aims to provide an update on the diagnostic accuracy in terms of estimating the specificity and sensitivity of RAT kits in patients with suspected COVID-19.

## 2. Methods

### 2.1. Study Protocol and Guideline

Based on the current literature, this systematic review and meta-analysis on the diagnostic accuracy of the available RAT kits for COVID-19 diagnosis was undertaken according to the PRISMA guideline [15]. The protocol of this study was registered in the PROSPERO database (CRD42021231432).

### 2.2. Eligibility Criteria

As the objective was to investigate the pooled sensitivity and specificity of the available RAT kits detecting SARS-CoV-2, we included studies in which RAT kits were used to identify SARS-CoV-2 to confirm COVID-19. Original studies without restricting study design or language were included. Review articles, opinions, case reports, news, press releases, blogs and data from websites were not considered eligible.

### 2.3. Search Strategies

Based on the eligibility criteria, we searched online databases such as PubMed, Google Scholar, Scopus, and Web of Science to identify studies of our interest published between 1 January 2020 and 13 January 2021. The following keywords were used to search different databases: COVID-19, SARS-CoV-2, coronavirus, nCoV, antigen, detection, diagnostic, diagnosis, test, testing, assay, assays and combined with appropriate Boolean operators (Table S1). Additionally, the reference lists of the included studies were also reviewed to identify any potentially eligible studies. EndNote X8 software (Clarivate Analytics, Philadelphia, PA, USA) was used to identify and exclude duplicate studies.

### 2.4. Study Selection

Three authors (S.S.K., N.H.H.N.H. and R.H.S.) independently screened the original pool of papers, then assessed their eligibility to be included in this meta-analysis using title, abstract, and full-text evaluation. Disagreements about whether a study should be included or excluded were discussed with the other authors (M.A.I. and Z.Z.D.) and resolved with acceptable consensus.

### 2.5. Data Extraction

Three authors (S.S.K., N.H.H.N.H. and R.H.S.) independently undertook the data extraction, and two authors (M.A.I. and Z.Z.D.) validated it. Initially, the data of the total number of positive and negative specimens confirmed by a reference standard followed by the number of positive and negative results of those same specimens were evaluated through RAT kits and they were extracted from each of the eligible studies. The major characteristics of the studies including the Study ID (last name of the first author and year of publication), location, total number of subjects, percentage of female subjects, the mean or median age of the participants, type of participants, specimen types, the test method of RAT kit used, percentages of positive samples detected by the reference standard (RT-PCR), ranges of Ct values of the reference standard and the manufacturer of the RAT kits were documented.

### 2.6. Quality Assessment

Two authors (S.S.K. and M.A.I.) independently assessed the quality of the included studies following the diagnostic test accuracy quality assessment tool of the Joanna Briggs Institute (JBI). To resolve the discrepancies, all authors took part in the discussion and resolved with consensus. If the total score was ≤49, 50–69, or ≥70%, the studies were classed as low-quality (high-risk of bias), moderate-quality (moderate-risk of bias), or high-quality (low risk of bias) [16]. To detect publication bias, funnel plots were constructed, and the Egger’s test was performed.

### 2.7. Data Analyses

A random-effects model with 95% confidence intervals (CIs) was used to analyse the overall pooled sensitivity and specificity of the RAT kit to detect COVID-19. *I*^2^ statistics were used to analyse heterogeneity among the included studies (*I*^2^ > 75% indicating substantial heterogeneity), followed by Cochran’s Q test to determine the significance of the heterogeneity (*p* < 0.05 was considered statistically significant). Furthermore, a Galbraith plot was generated to determine the outlier studies.

### 2.8. Subgroup and Sensitivity Analyses

Subgroup analyses were undertaken to investigate sensitivity and specificity based on symptomatic and asymptomatic patients, days of symptom onset, types of specimen, Ct values, countries, continents and manufacturers of RAT. To investigate the robustness of results and the possible source of heterogeneity, sensitivity analyses were performed through strategies such as excluding small studies (<100), excluding low- or moderate-quality studies, using a fixed-effects model, and excluding outlier studies. The analyses and plots were constructed by using the metaprop codes in the meta (version 4.15-1) and metafor (version 2.4-0) packages of R (version 3.6.3) in RStudio (RStudio, Inc., Boston, MA, USA) (version 1.3.1093).

## 3. Results

### 3.1. Study Selection

**Figure 1:**
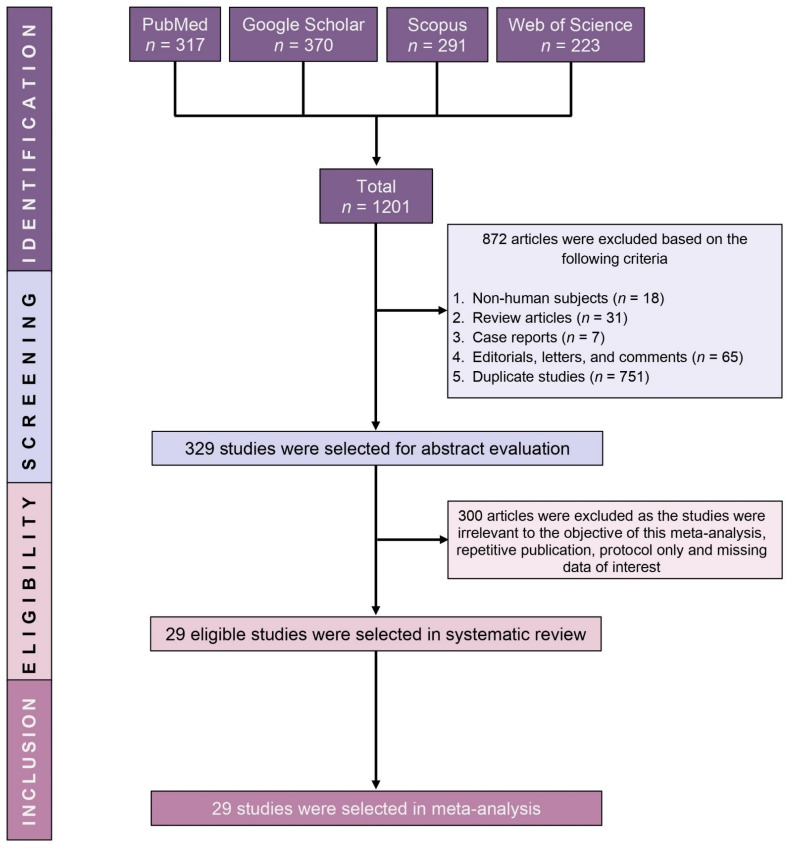
PRISMA flow diagram of study selection.

Primarily, based on the search strategies, a total of 1201 published articles were identified from the online databases. During the initial screening process, 872 articles including review articles (*n* = 31), case reports (*n* = 7), articles that included non-human subjects (*n* = 18), editorials, letters and comment (*n* = 65) and duplicate studies (*n* = 751) were excluded. From the remaining 329 studies, 300 studies did not comply with the objective of this meta-analysis (irrelevant to the objective of the meta-analysis, repetitive studies, protocols only or missing data of interest); hence regarded as ineligible. The remaining 29 studies fulfilled the eligibility criteria and were finally included in this meta-analysis (Figure 1).

### 3.2. Characteristics of the Included Studies

A total of 17,171 COVID-19 suspects, including RT-PCR-positive and negative participants who were further tested in RAT, were reported in this meta-analysis. Different types of specimen (i.e., nasopharyngeal swab, saliva, nasal swab, sputum, throat swab or endotracheal aspirates) were taken from suspected symptomatic or asymptomatic participants. The RT-PCR-positive participants had a wide range of Ct values. Although a total of 15 different RAT kits were assessed from 13 different manufacturers, the RAT test methods were based on either immunochromatographic (ICG) assay or fluorescence immunoassay (FIA). The studies were carried out in several countries encompassing five continents. Table 1 shows the detailed features of the studies we included.

### 3.3. Quality Assessment and Publication Bias

**Figure 2:**
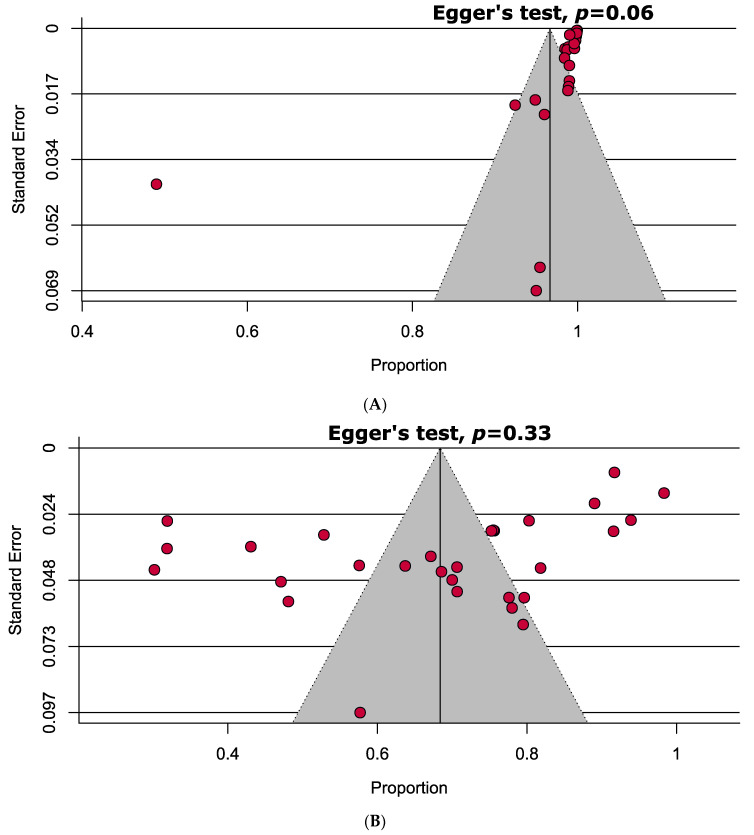
Funnel plots representing no evidence of significant publication bias estimating (**A**) specificity and (**B**) sensitivity of rapid antigen tests in confirming COVID-19.

The quality of each of the included studies was extensively examined using the JBI diagnostic accuracy checklist where the highest and lowest quality scores of the included studies were recorded as 88.8% (five studies) and 55.5% (two studies), respectively. Overall, there were no low-quality studies, 26.6% of high-quality, and 72.4% of moderate-quality studies (Table S2). There was no indication of substantial publication bias in the funnel plots evaluating the specificity and sensitivity of RAT kits to confirm SARS-CoV-2 (Figure 2).

### 3.4. Meta-Analysis

**Figure 3:**
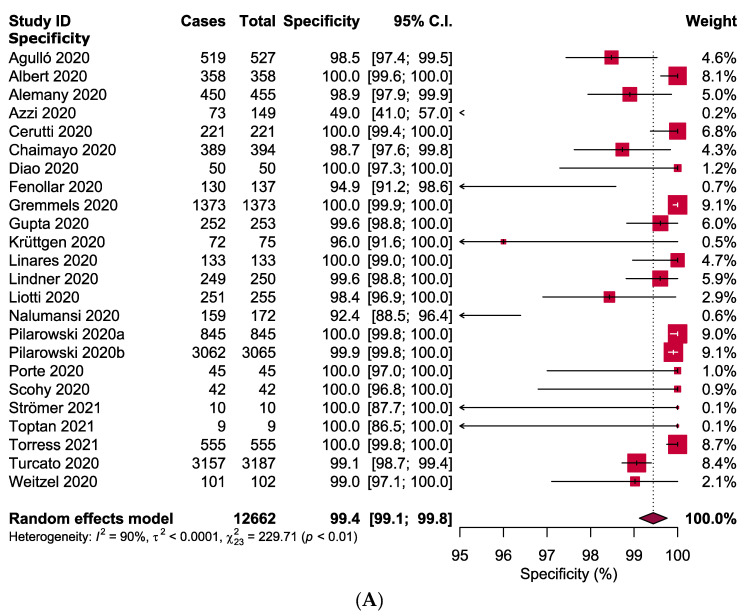

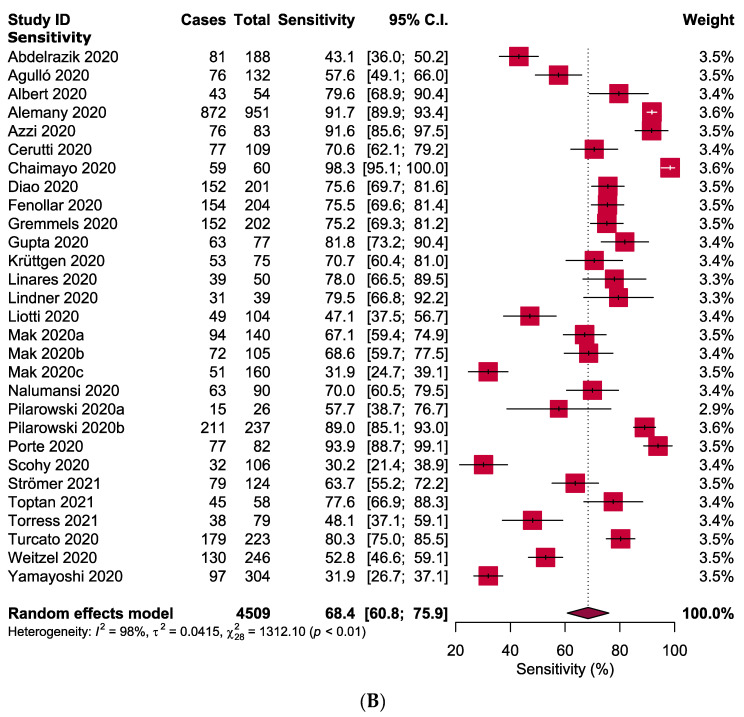
Forest plots representing estimating of (**A**) specificity and (**B**) sensitivity of rapid antigen tests in confirming COVID-19.

**Figure 4:**
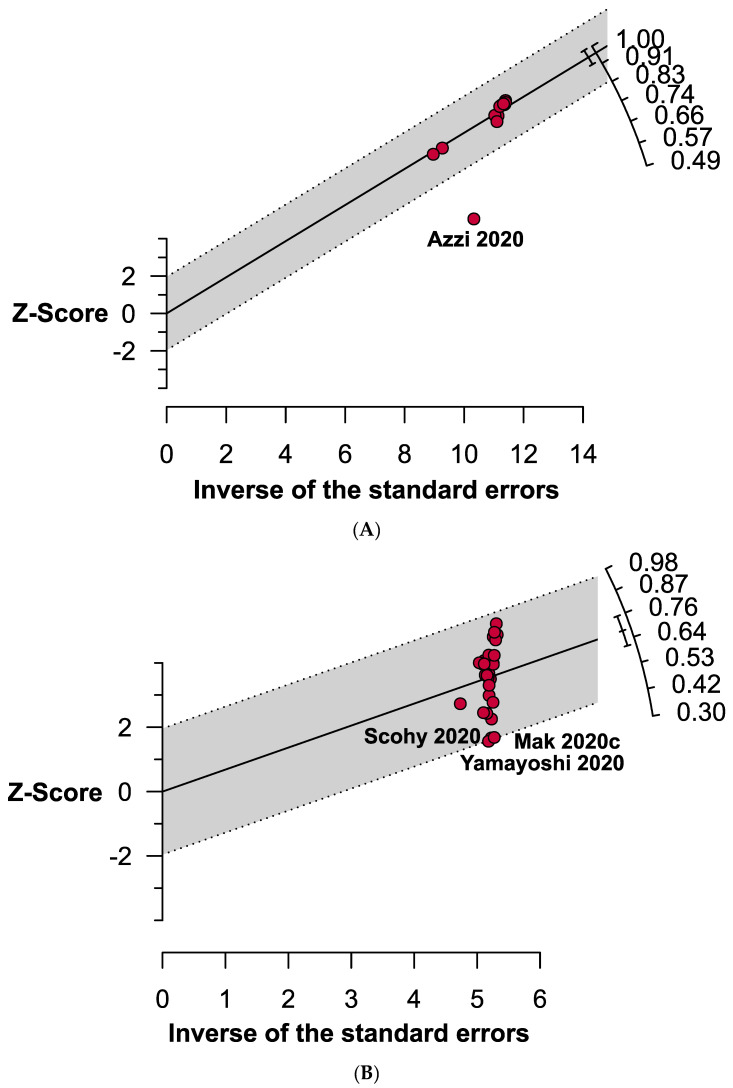
Galbraith plots representing outlier studies estimating (**A**) specificity and (**B**) sensitivity of rapid antigen tests in confirming COVID-19.

The overall pooled specificity and sensitivity of RAT were 99.4% (95% CI: 99.1–99.8; *I*^2^ = 90%) and 68.4% (95% CI: 60.8–75.9; *I*^2^ = 98%), respectively (Figure 3). Four outlier studies [20,33,38,44] were identified using the Galbraith plot (Figure 4). Except for the pooled specificity of subgroups based on specimen types (i.e., nasopharyngeal swab: 71.0% (95% CI: 14.1–100.1) and saliva: 80.7% (95% CI: 41.8–100.0)), the specificity did not vary in most of the subgroups and ranged between 99 and 100% (Table 2 and Figure S1). On the other hand, in subgroup analyses, the sensitivity for each subgroup was lower than the specificity. Each of the subgroups estimating the sensitivity had high levels of heterogeneity except for the subgroups defined by the onset of symptoms (<5 and >5 days; *I*^2^ = 0%) and Ct values (Ct values ≤20 and 36–40; *I*^2^ = 0%). When compared with asymptomatic patients (54.5%), the sensitivity of RAT kits was higher in symptomatic patients (78.5%). The sensitivity of the nasopharyngeal swab was higher (70.1%) than that of saliva (50.4%) and throat swab or saliva (38.4%). The CT indicates the number of cycles needed for the fluorescence signals to reach the threshold. Ct value for a particular gene (i.e., N, ORF, E, S or M) is inversely correlated with viral load in a targeted specimen [45,46]. Surprisingly, the sensitivity of RAT kits was shown to have an inverse relationship with the Ct values, which also corresponded with the sensitivity of subgroups based on the symptom onset days (Table 2 and Figure S1). The sensitivity of RAT kits dropped to 15.1% and 16.5% when Ct values were 31–35 and 36–40, respectively. On the other hand, the RAT kits had a lower sensitivity when used in the African (56.4%) and Asian (65.0%) populations compared with the European (70.0%) and American populations (74.1%). Kits from different manufacturers exhibited various sensitivity. Panbio^TM^ showed the highest sensitivity (75.1%) followed by Abbott BinaxNOW™ (74.8%), Standard™ (66.4%) and Biocredit (42.7%).

### 3.5. Sensitivity Analyses

To assess the range in outcomes, sensitivity analyses were performed removing small studies (*n* < 100), low- or moderate-quality studies, using a fixed-effects model instead of a random-effects model, and omitting outlier studies. Surprisingly, the specificity of RAT kits for each of the sensitivity analysis results remained very constant when compared to the total pooled specificity (99.4%), ranging from 99.4% to 99.9% (Table 3 and Figure S2). Using a fixed-effects model, the overall pooled sensitivity was enhanced to 79.9% as compared with the overall pooled sensitivity observed using the random-effects model (68.4%). However, when small (*n* < 100) studies, low- and moderate-quality studies, and outlier studies were excluded, the sensitivity of RAT kits was found to be 62.0%, 69.8%, and 71.2%, respectively, which was close to the overall pooled sensitivity (68.4%) (Table 3 and Figure S2).

## 4. Discussion

RT-PCR has been recognised as the diagnostic gold standard for COVID-19 diagnosis to date; however, it has several drawbacks, including false-negative and false-positive findings [47,48]. The rate of false-negative results was estimated to be about 67% in the first 4–5 days following the onset of symptoms. [48]. On the other hand, the reasons behind the false-positive results were contamination during specimen collection, contamination of reagents, PCR amplicons, cross-contamination of the sample, and cross-reaction with other genetic materials or viruses [47]. Even in a cohort study, chest CT was found to be more sensitive (88%) as compared with the diagnostic result of RT-PCR (59%) [49]. Additionally, RT-PCR is costly and requires well-equipped laboratories [47,48,50]. More importantly, the release of RT-PCR results in many instances may take longer than expected due to high sample volumes and a lack of technical support, leading to delayed management of patients and control of outbreaks. As a result, an emphasis on developing RAT kits was necessary to close the diagnostic gaps.

So far, several RAT kits have been developed by various manufacturers from multiple countries and evaluated independently by researchers. We observed that most of the RATs were developed based on either ICG assay or FIA. Although both the assays are generally based on the antibody–antigen interaction technique, for the detection, a signal generator (i.e., gold colloid) is used in ICG whereas a fluorescent molecule is bound to the detection antibody in FIA [51,52,53]. In this meta-analysis, we looked at the overall sensitivity and specificity of the available reported RAT kits. We found that although the overall pooled specificity (99.4%) was acceptable, the overall pooled sensitivity (68.4%) was not.

Except in a few subgroups where there was a lot of heterogeneity across the studies, overall, the specificity did not differ substantially in the subgroup analysis. The study results, on the other hand, differed considerably when it came to assessing the sensitivity. Interestingly, the sensitivity of RAT kits for symptomatic and asymptomatic patients was 78.5% and 54.5%, respectively, indicating that RAT kits can identify both kinds of patient, albeit at a lower detection rate in the latter patient group. However, early detection is critical for RAT, as the sensitivity maintained at 82.0% when the symptom onset came after less than 5 days but dropped to 75.1% after five days. In the case of RT-PCR, a similar association between days of symptom onset and correct diagnosis had been reported [48]. This correlation was further justified when we analysed the correlation between Ct values and sensitivity. Previously, an analogous observation was investigated where a negative correlation of Ct values of RT-PCR and sensitivity in just one antigen kit for a specimen was identified [54]. When we checked the overall scenario with the studies we included, we found a similar inverse correlation of Ct values and sensitivity. According to the Centers for Disease Control and Prevention (CDC), the higher Ct value (>33) can be considered as a non-contagious stage [55] which can justify the usage of RAT kits. However, early diagnosis using RAT kits can be suggested according to these observations. Again, among different specimen types, the nasopharyngeal swab was identified to be the best for SARS-CoV-2 identification using RAT kits. A recent study also suggested that the nasopharyngeal swab is more sensitive (89%) as compared with saliva (72%) to detect SARS-CoV-2 which confirms our findings [56].

In the current study, when the RAT sensitivity to detect SARS-CoV-2 in people from various continents and nations was examined, it was demonstrated that the sensitivity of RAT in the population of Europe and America was higher as compared to that of Asia and Africa. Because most RAT kits are manufactured in European countries, China, and Korea, the sensitivity may have diminished in Asia and Africa owing to issues during RAT kit transportation. As reported earlier, a repetitive freeze-thaw process during transportation can change the behaviour of proteins used in RAT kits [57]. This was further justified when a study by Gupta et al. [26] from India exhibited a much higher sensitivity (81.8% (95% CI: 73.2–90.4)) and the RAT kit used in this study was in fact manufactured in India. Hence, based on our findings, it can be suggested that local manufacturers or inventors should be prioritised for the RAT kit development and utilisation.

Further investigations are required to determine the correlation of different sample conditions such as transportation or storage with the sensitivity of RAT. Again, recent reports about different genetic and structural mutations of SARS-CoV-2 enhance the possibility to hamper the sensitivity of diagnostic tools [58,59]. Hence, researchers need to focus on the improvement of sensitivity of RAT without compromising the specificity to detect both the original and mutated variants of SARS-CoV-2. Results of this meta-analysis support the recommendations of the Infectious Diseases Society of America Guidelines on the Diagnosis of COVID-19 that the sensitivity of RAT kits is highly correlated with viral load, symptoms, and the timing of the test in relation to the onset of symptoms [60]. This meta-analysis also shows that the sensitivity of RAT kits differs depending on the manufacturer and the country in which the kits are produced.

## 5. Conclusions

To summarise, the use of RAT kits is largely recommended for the early detection of patients suspected of having COVID-19, particularly in densely populated and isolated locations with limited resources and laboratory equipment. It may also be useful for prompt diagnosis in central city or rural areas. However, the negative RAT samples may need to be further analysed using molecular tests to confirm the results, particularly when the symptoms of COVID-19 are present.

## Figures and Tables

**Table 1:** Major characteristics of the included studies.

Study ID [References]	Location	Total Subjects (% Female) Mean/Median Age	Type of Participants	Specimen Types	Positive Sample by RT-PCR (%)	Range of Ct Values	Testing Method	Rapid Antigen Test Kit (Manufacturer, Country)
Abdelrazik 2020 [17]	Egypt	310 (40.6, 42)	C-19 (*n* = 160) and HCW + CC (*n* = 150)	NPS	60.6	15.8–32.3	ICG	Biocredit COVID-19 Ag Detection Kit (RapiGEN, Korea)
Agulló 2020 [18]	Spain	659 (56.4, 38)	SS (*n* = 394) and CC (*n* = 265)	NS, S	20.0	14.0–33.0 (IQR)	ICG	Panbio™ COVID-19 Ag-RTD (Abbott Diagnostics, Germany)
Albert 2020 [12]	Spain	412 (58.0, 31)	SS (*n* = 412)	NPS	13.1	≤25–≤34	ICG	Panbio™ COVID-19 Ag-RTD (Abbott Diagnostics, Germany)
Alemany 2020 [19]	Spain	1406 (NR, 40)	SS (*n* = 446), CC (*n* = 473) and GS (*n* = 487)	NPS, NMT	67.6	19.7–27.3 (IQR)	ICG	Panbio™ COVID-19 Ag-RTD (Abbott Diagnostics, Germany)
Azzi 2020 ^†^ [20]	Italy	122 (67.2, 54)	C-19, SS and HCW	NPS, S	23.8	NR	ICG	In house
Cerutti 2020 [21]	Italy	185 (NR, 45)	SS (*n* = 185)	NPS	56.2	12.3–38.1	ICG	Standard™ Q COVID-19 Ag kit (SD Biosensor, Korea)
		145 (NR, 36)	T (*n* = 145)		3.4			
Chaimayo 2020 [22]	Thailand	454 (56.2, 58)	CC, SS, T and POS.	NPS, TS, EA	13.2	10.4–35.0	ICG	Standard™ Q COVID-19 Ag kit (SD Biosensor, Korea)
Diao 2020 ^†^ [23]	China	251 (51.4, 40)	SS (*n* = 251)	NPS	80.1	≤37.0–≤40.0	FIA	In house
Fenollar 2020 [24]	France	341 (NR, NR)	SS (*n* = 182) and CC (*n* = 159)	NPS	59.8	9.0–34.0	ICG	Panbio™ COVID-19 Ag-RTD (Abbott Diagnostics, Germany)
Gremmels 2020 ^†^ [25]	The Netherlands	1367 (61.7, 36)	GS (*n* = 1367)	NPS, TS	10.2	<32.0–≥32.0	ICG	Panbio™ COVID-19 Ag-RTD (Abbott Diagnostics, USA)
	Aruba	208 (NR, NR)	GS (*n* = 208)		30.3			
Gupta 2020 ^†^ [26]	India	330 (30.0, 34)	SS (*n* = 204) and CC (*n* = 126)	NS, TS	23.3	10.0–35.4	ICG	Standard™ Q COVID-19 Ag kit (SD Biosensor, India)
Krüttgen 2020 [27]	Germany	150 (NR, NR) ^#^	C-19 (*n* = 75) and non-C-19 (*n* = 75)	NPS	50.0	<25.0–≥35.0	ICG	SARS-CoV-2 Rapid Antigen Test (Roche, Switzerland)
Linares 2020 [28]	Spain	255 (51.4, 46 *)	SS, CC and AS	NPS	23.5	<25.0–<40.0	ICG	Panbio™ COVID-19 Ag-RTD (Abbott Diagnostics, Germany)
Lindner 2020 ^†^ [29]	Germany	287 (42.9, 35)	SS	NS **	13.5	17.3–≥35.5	ICG	Standard™ Q COVID-19 Ag kit (SD Biosensor, Korea)
Liotti 2020 [30]	Italy	359 (NR, NR) ^#^	C-19 (*n* = 104) and non-C-19 (*n* = 255)	NPS	29.0	15.3–39.7	FIA	Standard™ F COVID-19 Ag (SD Biosensor, Korea)
Mak 2020a [31]	Hong Kong	280 (NR, NR) ^#^	C-19 (*n* = 280)	S, NPS, NPA, TS	100.0	<18.6–>28.7	ICG	COVID-19 Ag Respi-Strip (Coris Bioconcept, Belgium)
								NADAL COVID-19 Ag Test (Nal Von Minden, Germany)
								Standard™ Q COVID-19 Ag kit (SD Biosensor, Korea)
Mak 2020b [32]	Hong Kong	105 (NR, NR) ^#^	C-19 (*n* = 105)	NPS, TS, S	100.0	<18.6–>28.7	ICG	Panbio™ COVID-19 Ag-RTD (Abbott Diagnostics, Germany)
								Standard™ Q COVID-19 Ag kit (SD Biosensor, Korea)
Mak 2020c [33]	Hong Kong	160 (NR, NR) ^#^	C-19 (*n* = 160)	S, NPS, TS, NPA, SP	100.0	<18.6–>28.7	ICG	Biocredit COVID-19 Ag Detection Kit, (RapiGEN, Korea)
Nalumansi 2020 [34]	Uganda	262 (10.7, 34)	SS (*n* = 136) and AS (*n* = 124)	NPS	34.4	<29–39	ICG	Standard™ Q COVID-19 Ag kit (SD Biosensor, Korea)
Pilarowski 2020a [35]	USA	878 (46.0, NR)	GS (*n* = 878)	NS	3.0	28.8–30.3	ICG	Abbott BinaxNOW™ COVID-19 Ag (Abbott Diagnostics, USA)
Pilarowski 2020b [36]	USA	3302 (45.4, NR)	GS (*n* = 3302)	NS	7.2	<30.0–<35.0	ICG	Abbott BinaxNOW™ COVID-19 Ag (Abbott Diagnostics, USA)
Porte 2020 ^†^ [37]	Chile	127 (46.5, 38)	SS + T + CC (*n* = 127)	NPS, OP	64.6	14.2–25.1 (IQR)	FIA	Bioeasy™ 2019-nCoV Ag RTK (Bioeasy Biotechnology, China)
Scohy 2020 [38]	Belgium	148 (56.8, 58)	SS (*n* = 148)	NPS	71.6	16.0–36.0	ICG	COVID-19 Ag Respi-Strip (Coris Bioconcept, Belgium)
Strömer 2021 ^‡^ [39]	Germany	134 (NR, NR) ^#^	C-19 (*n* = 124) and non-C-19 (*n* = 10)	NPS	92.5	17.0–37.0	ICG	NADAL COVID-19 Ag Test (Nal Von Minden, Germany)
								Panbio™ COVID-19 Ag-RTD (Abbott Diagnostics, Germany)
Toptan 2021 [40]	Germany	67 (NR, NR) ^#^	C-19 (*n* = 58) and Non-C-19 (*n* = 9)	OP, NS	86.6	18.7–40.0	ICG	RIDA^®^QUICK SARS-CoV-2 Ag test (R-Biopharm, Germany)
		70 (NR, NR)	GS (*n* = 70)	NS	45.7	18.0–35.9	ICG	RIDA^®^QUICK SARS-CoV-2 Ag test (R-Biopharm, Germany)
Torress 2021 [41]	Spain	634 (56.0, 37)	CC (*n* = 634)	NPS	12.4	≤20.0–>35.0	ICG	Panbio™ COVID-19 Ag-RTD (Abbott Diagnostics, Germany)
Turcato 2020 [42]	Italy	3410 (NR, NR)	SS (*n* = 991) and AS (*n* = 2419)	NR	6.5	NR	ICG	Standard™ Q COVID-19 Ag kit (SD Biosensor, Korea)
Weitzel 2020 ^†^ [43]	Chile	111 (55.0, 40)	SS (*n* = 111)	NPS	72.1	10.7–37.7	ICG	Biocredit COVID-19 Ag Detection Kit (RapiGEN, Korea)
							ICG	StrongStep COVID-19 Antigen test (Liming Bioproducts, China)
							FIA	Huaketai New Coronavirus (Savant Biotechnology, China)
							FIA	Bioeasy™ 2019-nCoV Ag RTK (Bioeasy Biotechnology, China)
Yamayoshi 2020 [44]	Japan	76 (NR, NR) ^#^	C-19 (*n* = 76)	S, TS, NS, NPS, SP, EA	100.0	18.8–36.0	ICG	Standard™ Q COVID-19 Ag kit (SD Biosensor, Korea)
								Espline^®^ SARS-CoV-2 (Fujirebio, Japan)
								QuickNavi™-COVID19 Ag (Denka Seiken, Japan)
								ImmunoAce SARS-CoV-2 (Tauns Laboratories, Japan)

RT-PCR: Reverse transcription polymerase chain reaction, NR: Not reported, ICG: Immunochromatographic assay, FIA: Fluorescence immunoassay, IQR: Interquartile range, C-19: Confirmed COVID-19 patient, SS: Symptomatic patient (suggestive of COVID-19), AS: Asymptomatic patient, CC: Asymptomatic contact with known COVID-19 or symptomatic patient, HCW: Healthcare workers, GS: General screening, T: Travelers, POS: Pre-operative screening, NPS: Nasopharyngeal swab, S: Saliva, NS: Nasal swab, TS: Throat swab, EA: Endotracheal aspirates, OP: Oro-pharyngeal swab, NPA: Nasopharyngeal aspirate, SP: Sputum, RTD: Rapid test device, NMT: Nasal mid-turbinate. ^#^ Archived samples of known RT-PCR result, ^†^ Reader-blinded study, ^‡^ With photo and intensity reader to support the reading, * Based on presumption mean calculation, ** Self-collected.

**Table 2:** Pooled specificity and sensitivity of SARS-CoV-2 detection using the rapid antigen test kit.

Subgroups		Pooled Specificity and Sensitivity (95% Cis) (%)	Number of Studies Analysed	Total Number of Patients	Heterogeneity	
					*I* ^2^	*p*-Value
**Specificity**						
Based on presence of symptoms	Symptomatic	99.1 (97.6–100.0)	4	1823	89%	<0.0001
	Asymptomatic	99.5 (98.6–100.0)	4	3280	92%	<0.0001
Based on symptom onset	Onset of symptoms <5 days	99.3 (98.8–99.8)	3	3537	34%	0.40
	Onset of symptoms >5 days	100.0 (99.0–100.0)	2	138	0%	0.48
Based on specimen types	Nasopharyngeal Swab	71.0 (14.1–100.1)	2	611	99%	<0.0001
	Saliva	80.7 (41.8–100.0)	2	547	97%	<0.0001
Based on continents	Asia	99.4 (98.7–100.0)	3	697	0%	0.43
	Europe	99.1 (98.6–99.7)	16	7736	93%	<0.0001
	North America	99.9 (99.8–100.0)	2	3910	0%	0.70
	South America	99.3 (97.7–100.0)	2	147	0%	0.95
Based on countries	Chile	99.3 (97.7–100.0)	2	147	0%	0.95
	Germany	99.5 (98.7–100.0)	4	344	0%	0.34
	Italy	94.0 (90.8–97.1)	4	3812	98%	<0.0001
	Spain	99.7 (99.2–100.0)	5	2028	67%	0.03
	USA	99.9 (99.8–100.0)	2	3910	0%	0.70
Based on kit manufacturers	Abbott BinaxNOW™	99.9 (99.8–100.0)	2	3910	0%	0.70
	Biocredit	99.0 (97.1–100.0)	1	102	NA	NA
	Panbio™	99.7 (99.4–100.0)	8	3547	72%	0.001
	Standard™	99.4 (98.8–100.0)	7	2100	74%	0.001
**Sensitivity**						
Based on presence of symptoms	Symptomatic	78.5 (61.2–95.9)	5	720	97%	<0.0001
	Asymptomatic	54.5 (24.3–84.7)	5	217	96%	<0.0001
Based on symptom onset	Onset of symptoms <5 days	82.0 (78.1–86.0)	4	357	0%	0.72
	Onset of symptoms >5 days	75.1 (64.8–85.4)	3	66	0%	0.43
Based on specimen types	Nasopharyngeal Swab	70.1 (54.1–86.1)	5	339	91%	<0.0001
	Saliva	50.4 (7.9–92.9)	3	284	99%	<0.0001
	Throat saliva or swab	38.4 (13.7–63.1)	4	193	93%	<0.0001
Based on Ct values	Ct value ≤20	98.8 (96.1–100.0)	5	108	0%	0.96
	Ct value 21–25	89.6 (80.1–99.0)	6	242	85%	<0.0001
	Ct value 26–30	55.4 (24.0–86.7)	7	323	98%	<0.0001
	Ct value 31–35	15.1 (4.5–25.7)	7	202	86%	<0.0001
	Ct value 36–40	16.5 (0.0–34.4)	3	17	0%	0.89
Based on continents	Africa	56.4 (30.0–82.7)	2	278	95%	<0.0001
	Asia	65.0 (42.3–87.8)	7	1047	99%	<0.0001
	Europe	70.0 (61.3–78.6)	16	2593	96%	<0.0001
	North America	74.8 (44.2–100.0)	2	263	90%	0.001
	South America	73.4 (33.2–100.0)	2	328	99%	<0.0001
Based on countries	Chile	73.4 (33.2–100.0)	2	328	99%	<0.0001
	Germany	72.8 (63.3–82.3)	8	815	91%	<0.0001
	Hong Kong	55.8 (31.3–80.3)	3	405	97%	<0.0001
	Italy	72.8 (56.7–88.8)	4	519	95%	<0.0001
	Spain	71.2 (52.6–89.8)	5	1266	97%	<0.0001
	USA	74.8 (44.2–100.0)	2	263	90%	0.001
Based on kit manufacturers	Abbott BinaxNOW™	74.8 (44.2–100.0)	2	263	90%	0.001
	Biocredit	42.7 (30.7–54.7)	3	594	89%	<0.0001
	Panbio™	75.1 (64.9–85.3)	9	1789	96%	<0.0001
	Standard™	66.4 (48.5–84.2)	8	634	97%	<0.0001

CIs: confidence intervals; NA: not applicable; Ct: cycle threshold.

**Table 3:** Sensitivity analyses.

Strategies of Sensitivity Analyses	Pooled Specificity and Sensitivity (95% Cis) (%)	Difference of Results	Number of Studies Analysed	Total Number of Subjects	Heterogeneity	
					*I* ^2^	*p*-Value
**Specificity**						
Excluding small studies (<100)	99.4 (99.1–99.8)	No change	18	12,431	93%	<0.0001
Excluding low- or moderate-quality studies	99.6 (99.1–100.0)	0.2% higher	6	2556	74%	<0.001
Using a fixed-effects model	99.9 (99.8–100.0)	0.5% higher	24	12,662	90%	<0.001
Excluding outlier studies	99.7 (99.5–99.9)	0.03% higher	23	12,513	71%	<0.001
**Sensitivity**						
Excluding small studies (<100)	62.0 (51.1–72.9)	6.4% lower	17	3736	99%	<0.0001
Excluding low- or moderate-quality studies	69.8 (56.7–82.9)	1.4% higher	8	937	96%	<0.0001
Using a fixed-effects model	79.9 (60.8–75.9)	11.5% higher	29	4509	98%	<0.0001
Excluding outlier studies	71.2 (64.8–77.6)	2.8% higher	27	4045	97%	<0.0001

CIs: confidence intervals

## Data Availability

The data presented in this study are available in the main text and supplementary materials.

## Supplementary Materials

The following are available online at https://www.mdpi.com/article/10.3390/jcm10163493/s1, Table S1: Search strategies, Table S2: Quality assessment of the included studies, Figure S1: Subgroup analyses based on the presence of symptoms, symptom onset, specimen types, Ct values, continents, countries, and kit manufacturers estimating the pooled specificity (A-AA) and sensitivity (AB-BJ) using rapid antigen test kit to diagnose SARS-CoV-2, Figure S2: Sensitivity analyses excluding small studies (<100), excluding low- or moderate-quality studies, using a fixed-effects model, and excluding outlier studies estimating polled specificity (A–D) and sensitivity (E–H) using a rapid antigen test kit to diagnose SARS-CoV-2.

## References

[1] Islam M.A., Kundu S., Alam S.S., Hossan T., Kamal M.A., Hassan R. (2021). Prevalence and characteristics of fever in adult and paediatric patients with coronavirus disease 2019 (COVID-19): A systematic review and meta-analysis of 17515 patients. PLoS ONE.

[2] Saniasiaya J., Islam M.A., Abdullah B. (2021). Prevalence of Olfactory Dysfunction in Coronavirus Disease 2019 (COVID-19): A Meta-analysis of 27,492 Patients. Laryngoscope.

[3] Saniasiaya J., Islam M.A., Abdullah B. (2020). Prevalence and Characteristics of Taste Disorders in Cases of COVID-19: A Meta-analysis of 29,349 Patients. Otolaryngol. Head Neck Surg..

[4] Islam M.A., Alam S.S., Kundu S., Hossan T., Kamal M.A., Cavestro C. (2020). Prevalence of Headache in Patients With Coronavirus Disease 2019 (COVID-19): A Systematic Review and Meta-Analysis of 14,275 Patients. Front. Neurol..

[5] Guan W.-J., Ni Z.-Y., Hu Y., Liang W.-H., Ou C.-Q., He J.-X., Liu L., Shan H., Lei C.-L., Hui D.S. (2020). Clinical characteristics of coronavirus disease 2019 in China. N. Engl. J. Med..

[6] Pfefferle S., Reucher S., Nörz D., Lütgehetmann M. (2020). Evaluation of a quantitative RT-PCR assay for the detection of the emerging coronavirus SARS-CoV-2 using a high throughput system. Eurosurveillance.

[7] LeBlanc J.J., Gubbay J.B., Li Y., Needle R., Arneson S.R., Marcino D., Charest H., Desnoyers G., Dust K., Fattouh R. (2020). Real-time PCR-based SARS-CoV-2 detection in Canadian laboratories. J. Clin. Virol..

[8] US Food and Drug Administration In Vitro Diagnostics EUAs. Updated January 14. https://www.fda.gov/medical-devices/coronavirus-disease-2019-covid-19-emergency-use-authorizations-medical-devices/vitro-diagnostics-euas.

[9] D’Cruz R.J., Currier A.W., Sampson V.B. (2020). Laboratory testing methods for novel severe acute respiratory syndrome-coronavirus-2 (SARS-CoV-2). Front. Cell Dev. Biol..

[10] Torres I., Poujois S., Albert E., Álvarez G., Colomina J., Navarro D. (2021). Point-of-care evaluation of a rapid antigen test (CLINITEST^Ⓡ^ Rapid COVID-19 Antigen Test) for diagnosis of SARS-CoV-2 infection in symptomatic and asymptomatic individuals. J. Infect..

[11] Peeling R.W., Olliaro P.L., Boeras D.I., Fongwen N. (2021). Scaling up COVID-19 rapid antigen tests: Promises and challenges. Lancet Infect. Dis..

[12] Albert E., Torres I., Bueno F., Huntley D., Molla E., Fernández-Fuentes M.Á., Martínez M., Poujois S., Forqué L., Valdivia A. (2020). Field evaluation of a rapid antigen test (Panbio™ COVID-19 Ag Rapid Test Device) for COVID-19 diagnosis in primary healthcare centres. Clin. Microbiol. Infect..

[13] Mina M.J., Parker R., Larremore D.B. (2020). Rethinking Covid-19 test sensitivity—A strategy for containment. N. Engl. J. Med..

[14] World Health Organization (2020). Antigen-Detection in the Diagnosis of SARS-CoV-2 Infection Using Rapid Immunoassays: Interim Guidance. https://www.who.int/publications/i/item/antigen-detection-in-the-diagnosis-of-sars-cov-2infection-using-rapid-immunoassays.

[15] Moher D., Liberati A., Tetzlaff J., Altman D.G., Group P. (2009). Preferred reporting items for systematic reviews and meta-analyses: The PRISMA statement. PLoS Med..

[16] Chia Y.C., Islam M.A., Hider P., Woon P.Y., Johan M.F., Hassan R., Ramli M. (2021). The Prevalence of TET2 Gene Mutations in Patients with BCR-ABL-Negative Myeloproliferative Neoplasms (MPN): A Systematic Review and Meta-Analysis. Cancers.

[17] Abdelrazik A.M., Elshafie S.M., Abdelaziz H.M. (2020). Potential Use of Antigen-Based Rapid Test for SARS-CoV-2 in Respiratory Specimens in Low-Resource Settings in Egypt for Symptomatic Patients and High-Risk Contacts. Lab. Med..

[18] Agulló V., Fernández-González M., de la Tabla V.O., Gonzalo-Jiménez N., García J.A., Masiá M., Gutiérrez F. (2020). Evaluation of the rapid antigen test Panbio COVID-19 in saliva and nasal swabs in a population-based point-of-care study. J. Infect..

[19] Alemany A., Baro B., Ouchi D., Rodó P., Ubals M., Corbacho-Monné M., Vergara-Alert J., Rodon J., Segalés J., Esteban C. (2020). Analytical and clinical performance of the panbio COVID-19 antigen-detecting rapid diagnostic test. J. Infect..

[20] Azzi L., Baj A., Alberio T., Lualdi M., Veronesi G., Carcano G., Ageno W., Gambarini C., Maffioli L., Di Saverio S. (2020). Rapid Salivary Test suitable for a mass screening program to detect SARS-CoV-2: A diagnostic accuracy study. J. Infect..

[21] Cerutti F., Burdino E., Milia M.G., Allice T., Gregori G., Bruzzone B., Ghisetti V. (2020). Urgent need of rapid tests for SARS CoV-2 antigen detection: Evaluation of the SD-Biosensor antigen test for SARS-CoV-2. J. Clin. Virol..

[22] Chaimayo C., Kaewnaphan B., Tanlieng N., Athipanyasilp N., Sirijatuphat R., Chayakulkeeree M., Angkasekwinai N., Sutthent R., Puangpunngam N., Tharmviboonsri T. (2020). Rapid SARS-CoV-2 antigen detection assay in comparison with real-time RT-PCR assay for laboratory diagnosis of COVID-19 in Thailand. Virol. J..

[23] Diao B., Wen K., Zhang J., Chen J., Han C., Chen Y., Wang S., Deng G., Zhou H., Wu Y. (2020). Accuracy of a nucleocapsid protein antigen rapid test in the diagnosis of SARS-CoV-2 infection. Clin. Microbiol. Infect..

[24] Fenollar F., Bouam A., Ballouche M., Fuster L., Prudent E., Colson P., Tissot-Dupont H., Million M., Drancourt M., Raoult D. (2020). Evaluation of the Panbio Covid-19 rapid antigen detection test device for the screening of patients with COVID-19. J. Clin. Microbiol..

[25] Gremmels H., Winkel B.M., Schuurman R., Rosingh A., Rigter N.A., Rodriguez O., Ubijaan J., Wensing A.M., Bonten M.J., Hofstra L.M. (2021). Real-life validation of the Panbio™ COVID-19 antigen rapid test (Abbott) in community-dwelling subjects with symptoms of potential SARS-CoV-2 infection. EClinicalMedicine.

[26] Gupta A., Khurana S., Das R., Srigyan D., Singh A., Mittal A., Singh P., Soneja M., Kumar A., Singh A.K. (2020). Rapid chromatographic immunoassay-based evaluation of COVID-19: A cross-sectional, diagnostic test accuracy study & its implications for COVID-19 management in India. Indian J. Med. Res..

[27] Krüttgen A., Cornelissen C.G., Dreher M., Hornef M.W., Imöhl M., Kleines M. (2021). Comparison of the SARS-CoV-2 Rapid antigen test to the real star Sars-CoV-2 RT PCR kit. J. Virol. Methods.

[28] Linares M., Pérez-Tanoira R., Carrero A., Romanyk J., Pérez-García F., Gómez-Herruz P., Arroyo T., Cuadros J. (2020). Panbio antigen rapid test is reliable to diagnose SARS-CoV-2 infection in the first 7 days after the onset of symptoms. J. Clin. Virol..

[29] Lindner A.K., Nikolai O., Kausch F., Wintel M., Hommes F., Gertler M., Krüger L.J., Gaeddert M., Tobian F., Lainati F. (2020). Head-to-head comparison of SARS-CoV-2 antigen-detecting rapid test with self-collected anterior nasal swab versus professional-collected nasopharyngeal swab. Eur. Respir. J..

[30] Liotti F.M., Menchinelli G., Lalle E., Palucci I., Marchetti S., Colavita F., La Sorda M., Sberna G., Bordi L., Sanguinetti M. (2020). Performance of a novel diagnostic assay for rapid SARS-CoV-2 antigen detection in nasopharynx samples. Clin. Microbiol. Infect..

[31] Mak G.C., Lau S.S., Wong K.K., Chow N.L., Lau C., Lam E.T., Chan R.C., Tsang D.N. (2020). Analytical sensitivity and clinical sensitivity of the three rapid antigen detection kits for detection of SARS-CoV-2 virus. J. Clin. Virol..

[32] Mak G.C., Lau S.S., Wong K.K., Chow N.L., Lau C., Lam E.T., Chan R.C., Tsang D.N. (2021). Evaluation of rapid antigen detection kit from the WHO Emergency Use List for detecting SARS-CoV-2. J. Clin. Virol..

[33] Mak G.C., Cheng P.K., Lau S.S., Wong K.K., Lau C., Lam E.T., Chan R.C., Tsang D.N. (2020). Evaluation of rapid antigen test for detection of SARS-CoV-2 virus. J. Clin. Virol..

[34] Nalumansi A., Lutalo T., Kayiwa J., Watera C., Balinandi S., Kiconco J., Nakaseegu J., Olara D., Odwilo E., Serwanga J. (2021). Field evaluation of the performance of a SARS-CoV-2 antigen rapid diagnostic test in Uganda using nasopharyngeal samples. Int. J. Infect. Dis..

[35] Pilarowski G., Lebel P., Sunshine S., Liu J., Crawford E., Marquez C., Rubio L., Chamie G., Martinez J., Peng J. (2020). Performance characteristics of a rapid SARS-CoV-2 antigen detection assay at a public plaza testing site in San Francisco. medRxiv.

[36] Pilarowski G., Marquez C., Rubio L., Peng J., Martinez J., Black D., Chamie G., Jones D., Jacobo J., Tulier-Laiwa V. (2020). Field performance and public health response using the BinaxNOW TM Rapid SARS-CoV-2 antigen detection assay during community-based testing. Clin. Infect. Dis..

[37] Porte L., Legarraga P., Vollrath V., Aguilera X., Munita J.M., Araos R., Pizarro G., Vial P., Iruretagoyena M., Dittrich S. (2020). Evaluation of a novel antigen-based rapid detection test for the diagnosis of SARS-CoV-2 in respiratory samples. Int. J. Infect. Dis..

[38] Scohy A., Anantharajah A., Bodéus M., Kabamba-Mukadi B., Verroken A., Rodriguez-Villalobos H. (2020). Low performance of rapid antigen detection test as frontline testing for COVID-19 diagnosis. J. Clin. Virol..

[39] Strömer A., Rose R., Schäfer M., Schön F., Vollersen A., Lorentz T., Fickenscher H., Krumbholz A. (2021). Performance of a Point-of-Care Test for the Rapid Detection of SARS-CoV-2 Antigen. Microorganisms.

[40] Toptan T., Eckermann L., Pfeiffer A.E., Hoehl S., Ciesek S., Drosten C., Corman V.M. (2021). Evaluation of a SARS-CoV-2 rapid antigen test: Potential to help reduce community spread?. J. Clin. Virol..

[41] Torres I., Poujois S., Albert E., Colomina J., Navarro D. (2021). Evaluation of a rapid antigen test (Panbio™ COVID-19 Ag rapid test device) for SARS-CoV-2 detection in asymptomatic close contacts of COVID-19 patients. Clin. Microbiol. Infect..

[42] Turcato G., Zaboli A., Pfeifer N., Ciccariello L., Sibilio S., Tezza G., Ausserhofer D. (2020). Clinical application of a rapid antigen test for the detection of SARS-CoV-2 infection in symptomatic and asymptomatic patients evaluated in the emergency department: A preliminary report. J. Infect..

[43] Weitzel T., Legarraga P., Iruretagoyena M., Pizarro G., Vollrath V., Araos R., Munita J.M., Porte L. (2021). Comparative evaluation of four rapid SARS-CoV-2 antigen detection tests using universal transport medium. Travel Med. Infect. Dis..

[44] Yamayoshi S., Sakai-Tagawa Y., Koga M., Akasaka O., Nakachi I., Koh H., Maeda K., Adachi E., Saito M., Nagai H. (2020). Comparison of Rapid Antigen Tests for COVID-19. Viruses.

[45] Oishee M.J., Ali T., Jahan N., Khandker S.S., Haq M.A., Khondoker M.U., Sil B.K., Lugova H., Krishnapillai A., Abubakar A.R. (2021). COVID-19 pandemic: Review of contemporary and forthcoming detection tools. Infect. Drug Resist..

[46] Rao S.N., Manissero D., Steele V.R., Pareja J. (2020). A narrative systematic review of the clinical utility of cycle threshold values in the context of COVID-19. Infect. Dis. Ther..

[47] Surkova E., Nikolayevskyy V., Drobniewski F. (2020). False-positive COVID-19 results: Hidden problems and costs. Lancet Respir. Med..

[48] Kucirka L.M., Lauer S.A., Laeyendecker O., Boon D., Lessler J. (2020). Variation in false-negative rate of reverse transcriptase polymerase chain reaction–based SARS-CoV-2 tests by time since exposure. Ann. Intern. Med..

[49] Ai T., Yang Z., Hou H., Zhan C., Chen C., Lv W., Tao Q., Sun Z., Xia L. (2020). Correlation of chest CT and RT-PCR testing for coronavirus disease 2019 (COVID-19) in China: A report of 1014 cases. Radiology.

[50] Sil B.K., Jahan N., Haq M.A., Oishee M.J., Ali T., Khandker S.S., Kobatake E., Mie M., Khondoker M.U., Jamiruddin M.R. (2021). Development and performance evaluation of a rapid in-house ELISA for retrospective serosurveillance of SARS-CoV-2. PLoS ONE.

[51] Paek S.-H., Lee S.-H., Cho J.-H., Kim Y.-S. (2000). Development of rapid one-step immunochromatographic assay. Methods.

[52] Odell I.D., Cook D. (2013). Immunofluorescence techniques. J. Investig. Dermatol..

[53] Sil B.K., Jamiruddin M.R., Haq M.A., Khondoker M.U., Jahan N., Khandker S.S., Ali T., Oishee M.J., Kaitsuka T., Mie M. (2021). AuNP Coupled Rapid Flow-Through Dot-Blot Immuno-Assay for Enhanced Detection of SARS-CoV-2 Specific Nucleocapsid and Receptor Binding Domain IgG. Int. J. Nanomed..

[54] Lanser L., Bellmann-Weiler R., Öttl K.-W., Huber L., Griesmacher A., Theurl I., Weiss G. (2020). Evaluating the clinical utility and sensitivity of SARS-CoV-2 antigen testing in relation to RT-PCR Ct values. Infection.

[55] Centers for Disease Control and Prevention (2021). Interim Guidance on Ending Isolation and Precautions for Adults with COVID-19. https://www.cdc.gov/coronavirus/2019-ncov/hcp/duration-isolation.html.

[56] Jamal A.J., Mozafarihashjin M., Coomes E., Powis J., Li A.X., Paterson A., Anceva-Sami S., Barati S., Crowl G., Faheem A. (2021). Sensitivity of nasopharyngeal swabs and saliva for the detection of severe acute respiratory syndrome coronavirus 2. Clin. Infect. Dis..

[57] Zhao L., Li L., Liu G.-q., Chen L., Liu X., Zhu J., Li B. (2013). Effect of freeze–thaw cycles on the molecular weight and size distribution of gluten. Food Res. Int..

[58] Adnan N., Khondoker M.U., Rahman M.S., Ahmed M.F., Sharmin S., Sharif N., Azmuda N., Akter S., Nahar S., Mou T.J. (2021). Coding-complete genome sequences and mutation profiles of nine SARS-CoV-2 strains detected from COVID-19 patients in Bangladesh. Microbiol. Resour. Announc..

[59] Singh J., Samal J., Kumar V., Sharma J., Agrawal U., Ehtesham N.Z., Sundar D., Rahman S.A., Hira S., Hasnain S.E. (2021). Structure-function analyses of new SARS-CoV-2 variants B. 1.1. 7, B. 1.351 and B. 1.1. 28.1: Clinical, diagnostic, therapeutic and public health implications. Viruses.

[60] Hanson K.E., Altayar O., Caliendo A.M., Arias C.A., Englund J.A., Hayden M.K., Lee M.J., Loeb M., Patel R., El Alayli A. (2021). The Infectious Diseases Society of America Guidelines on the Diagnosis of COVID-19: Antigen Testing. Clin. Infect. Dis..

