# Supplementary material for: Diagnostic Accuracy of Rapid Antigen Test Kits for Detecting SARS-CoV-2: A Systematic Review and Meta-Analysis of 17,171 Suspected COVID-19 Patients
Source: J Clin Med. 2021 Aug 8;10(16):3493. doi: 10.3390/jcm10163493 (PMC8397079; doi:10.3390/jcm10163493)
Supplement: Supplementary file 1 [file jcm-10-03493-s001.zip › Supplementary Files/Figure S1_Subgroup analyses.pdf]

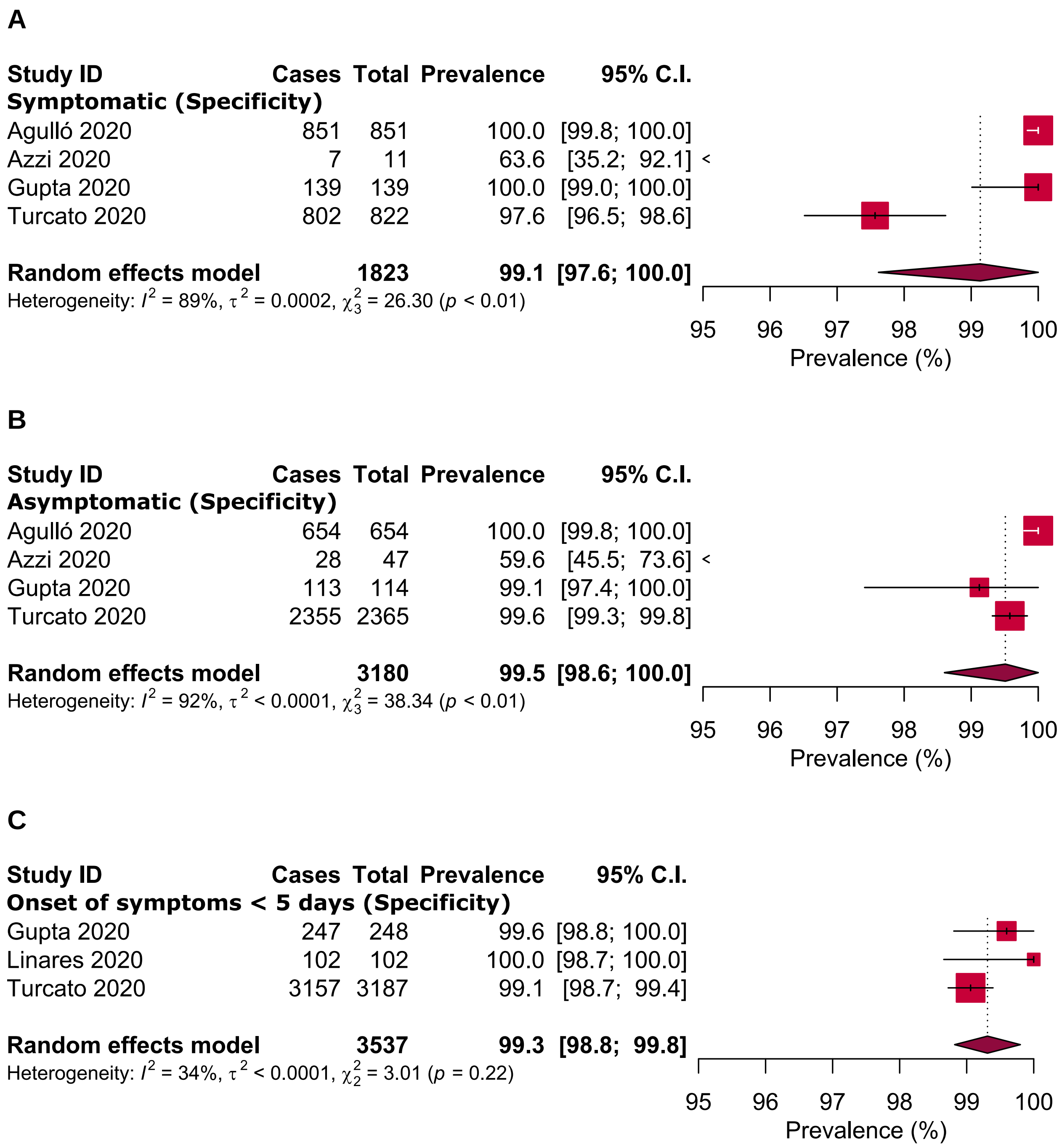
A
Study ID Cases Total Prevalence 95% C.I.
Symptomatic (Specificity)
Agulló 2020 851 851 100.0 [99.8; 100.0]
Azzi 2020 7 11 63.6 [35.2; 92.1] <
Gupta 2020 139 139 100.0 [99.0; 100.0]
Turcato 2020 802 822 97.6 [96.5; 98.6]
Random effects model 1823 99.1 [97.6; 100.0]
Heterogeneity: I^2 = 89%, τ^2 = 0.0002, χ^2_3 = 26.30 (p < 0.01)
95 96 97 98 99 100
Prevalence (%)
B
Study ID Cases Total Prevalence 95% C.I.
Asymptomatic (Specificity)
Agulló 2020 654 654 100.0 [99.8; 100.0]
Azzi 2020 28 47 59.6 [45.5; 73.6] <
Gupta 2020 113 114 99.1 [97.4; 100.0]
Turcato 2020 2355 2365 99.6 [99.3; 99.8]
Random effects model 3180 99.5 [98.6; 100.0]
Heterogeneity: I^2 = 92%, τ^2 < 0.0001, χ^2_3 = 38.34 (p < 0.01)
95 96 97 98 99 100
Prevalence (%)
C
Study ID Cases Total Prevalence 95% C.I.
Onset of symptoms < 5 days (Specificity)
Gupta 2020 247 248 99.6 [98.8; 100.0]
Linares 2020 102 102 100.0 [98.7; 100.0]
Turcato 2020 3157 3187 99.1 [98.7; 99.4]
Random effects model 3537 99.3 [98.8; 99.8]
Heterogeneity: I^2 = 34%, τ^2 < 0.0001, χ^2_2 = 3.01 (p = 0.22)
95 96 97 98 99 100
Prevalence (%)

D

Study ID	Cases	Total	Prevalence	95% C.I.
**Onset of symptoms > 5 days (Specificity)**				
Gupta 2020	5	5	100.0	[77.9; 100]
Linares 2020	133	133	100.0	[99.0; 100]
**Random effects model**		**138**	**100.0**	**[99.0; 100]**

Heterogeneity: $I^2 = 0\%$, $\tau^2 = 0$, $\chi_1^2 = 0.00$ ($p = 1.00$)

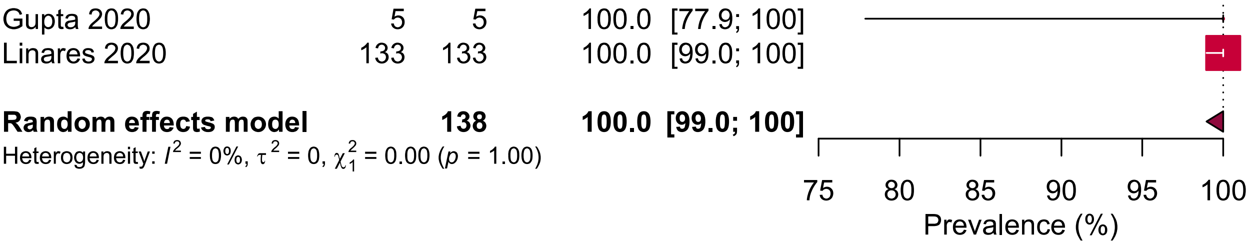

E

Study ID	Cases	Total	Prevalence	95% C.I.
**Nasopharyngeal Swab (Specificity)**				
Agulló 2020	519	520	99.8	[99.4; 100.0]
Azzi 2020	38	91	41.8	[31.6; 51.9]
**Random effects model**		**611**	**71.0**	**[14.1; 100.0]**

Heterogeneity: $I^2 = 99\%$, $\tau^2 = 0.1671$, $\chi_1^2 = 125.91$ ($p < 0.01$)

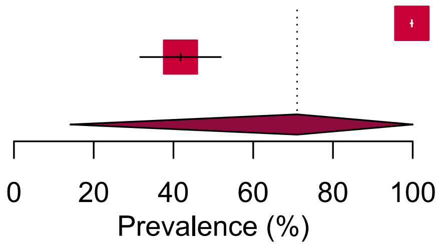

F

Study ID	Cases	Total	Prevalence	95% C.I.
**Saliva (Specificity)**				
Agulló 2020	489	489	100.0	[99.7; 100.0]
Azzi 2020	35	58	60.3	[47.8; 72.9]
**Random effects model**		**547**	**80.7**	**[41.8; 100.0]**

Heterogeneity: $I^2 = 97\%$, $\tau^2 = 0.0766$, $\chi_1^2 = 38.10$ ($p < 0.01$)

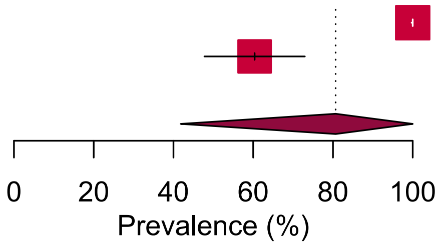

G

Study ID	Cases	Total	Prevalence	95% C.I.
**Africa (Specificity)**				
Nalumansi 2020	159	172	92.4	[88.5; 96.4]

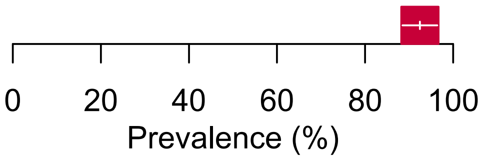

H

Study ID	Cases	Total	Prevalence	95% C.I.
**Asia (Specificity)**				
Chaimayo 2020	389	394	98.7	[97.6; 99.8]
Diao 2020	50	50	100.0	[97.3; 100.0]
Gupta 2020	252	253	99.6	[98.8; 100.0]
**Random effects model**		**697**	**99.4**	**[98.7; 100.0]**

Heterogeneity: $I^2 = 0\%$, $\tau^2 = 0$, $\chi^2_2 = 1.84$ ($p = 0.40$)

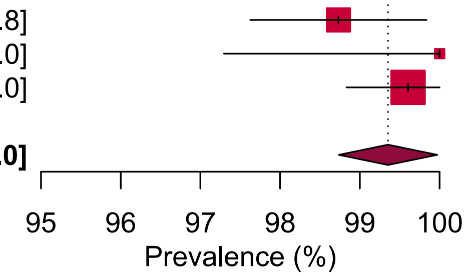

I

Study ID	Cases	Total	Prevalence	95% C.I.
**Europe (Specificity)**				
Agulló 2020	519	527	98.5	[97.4; 99.5]
Albert 2020	358	358	100.0	[99.6; 100.0]
Alemany 2020	450	455	98.9	[97.9; 99.9]
Azzi 2020	73	149	49.0	[41.0; 57.0]
Cerutti 2020	221	221	100.0	[99.4; 100.0]
Fenollar 2020	130	137	94.9	[91.2; 98.6]
Gremmels 2020	1373	1373	100.0	[99.9; 100.0]
Krüttgen 2020	72	75	96.0	[91.6; 100.0]
Linares 2020	133	133	100.0	[99.0; 100.0]
Lindner 2020	249	250	99.6	[98.8; 100.0]
Liotti 2020	251	255	98.4	[96.9; 100.0]
Scohy 2020	42	42	100.0	[96.8; 100.0]
Strömer 2021	10	10	100.0	[87.7; 100.0]
Toptan 2021	9	9	100.0	[86.5; 100.0]
Torress 2021	555	555	100.0	[99.8; 100.0]
Turcato 2020	3157	3187	99.1	[98.7; 99.4]
**Random effects model**		**7736**	**99.1**	**[98.6; 99.7]**

Heterogeneity: $I^2 = 93\%$, $\tau^2 < 0.0001$, $\chi^2_{15} = 208.95$ ($p < 0.01$)

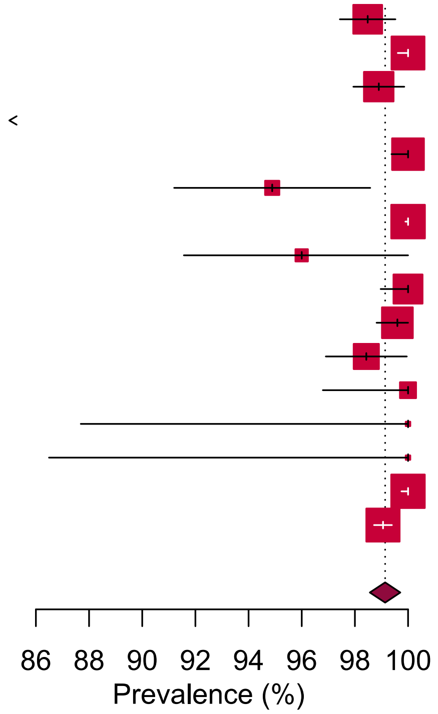

J

Study ID	Cases	Total	Prevalence	95% C.I.
**North America (Specificity)**				
Pilarowski 2020a	845	845	100.0	[99.8; 100]
Pilarowski 2020b	3062	3065	99.9	[99.8; 100]
**Random effects model**		**3910**	**99.9**	**[99.8; 100]**

Heterogeneity: $I^2 = 0\%$, $\tau^2 = 0$, $\chi^2_1 = 0.94$ ($p = 0.33$)

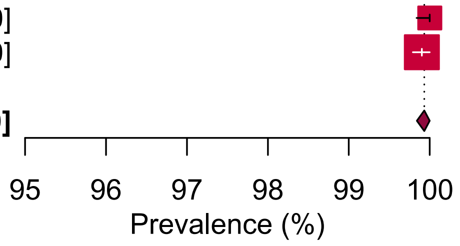

K

Study ID	Cases	Total	Prevalence	95% C.I.
**South America (Specificity)**				
Porte 2020	45	45	100.0	[97.0; 100]
Weitzel 2020	101	102	99.0	[97.1; 100]
**Random effects model**		**147**	**99.3**	**[97.7; 100]**

Heterogeneity: $I^2 = 0\%$, $\tau^2 = 0$, $\chi_1^2 = 0.29$ ($p = 0.59$)

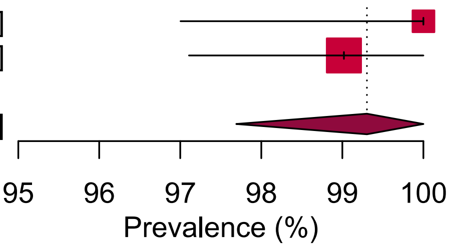

L

Study ID	Cases	Total	Prevalence	95% C.I.
**Belgium (Specificity)**				
Scohy 2020	42	42	100.0	[96.8; 100]

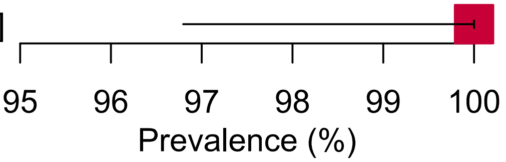

M

Study ID	Cases	Total	Prevalence	95% C.I.
**Chile (Specificity)**				
Porte 2020	45	45	100.0	[97.0; 100]
Weitzel 2020	101	102	99.0	[97.1; 100]
**Random effects model**		**147**	**99.3**	**[97.7; 100]**

Heterogeneity: $I^2 = 0\%$, $\tau^2 = 0$, $\chi_1^2 = 0.29$ ($p = 0.59$)

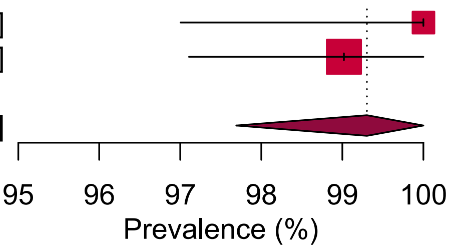

N

Study ID	Cases	Total	Prevalence	95% C.I.
**China (Specificity)**				
Diao 2020	50	50	100.0	[97.3; 100]

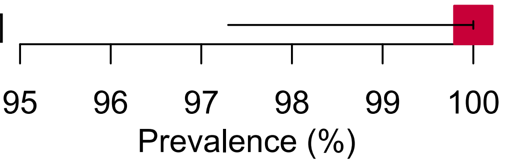

O

Study ID	Cases	Total	Prevalence	95% C.I.
**France (Specificity)**				
Fenollar 2020	130	137	94.9	[91.2; 98.6]

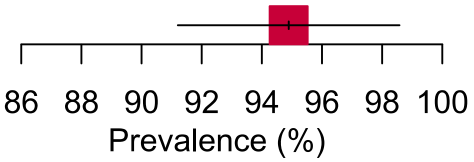

P

Study ID	Cases	Total	Prevalence	95% C.I.
**Germany (Specificity)**				
Krüttgen 2020	72	75	96.0	[91.6; 100]
Lindner 2020	249	250	99.6	[98.8; 100]
Strömer 2021	10	10	100.0	[87.7; 100]
Toptan 2021	9	9	100.0	[86.5; 100]
**Random effects model**		**344**	**99.5**	**[98.7; 100]**

Heterogeneity: $I^2 = 0\%$, $\tau^2 = 0$, $\chi^2_3 = 2.47$ ($p = 0.48$)

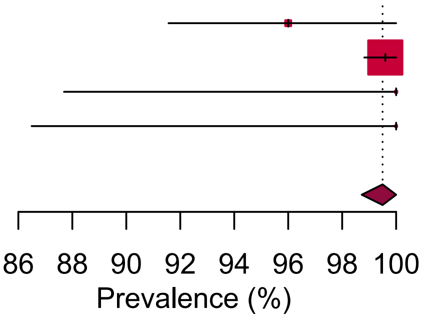

Q

Study ID	Cases	Total	Prevalence	95% C.I.
**India (Specificity)**				
Gupta 2020	252	253	99.6	[98.8; 100]

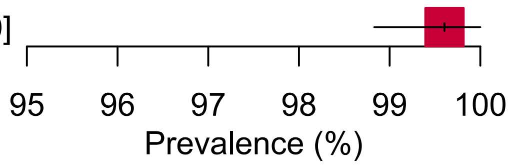

R

Study ID	Cases	Total	Prevalence	95% C.I.
**Italy (Specificity)**				
Azzi 2020	73	149	49.0	[41.0; 57.0]
Cerutti 2020	221	221	100.0	[99.4; 100.0]
Liotti 2020	251	255	98.4	[96.9; 100.0]
Turcato 2020	3157	3187	99.1	[98.7; 99.4]
**Random effects model**		**3812**	**94.0**	**[90.8; 97.1]**

Heterogeneity: $I^2 = 98\%$, $\tau^2 = 0.0009$, $\chi^2_3 = 158.25$ ($p < 0.01$)

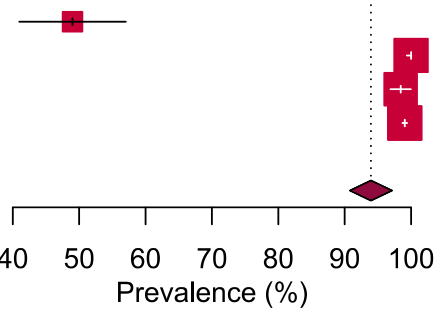

S

Study ID	Cases	Total	Prevalence	95% C.I.
**Netherlands (Specificity)**				
Gremmels 2020	1373	1373	100.0	[99.9; 100]

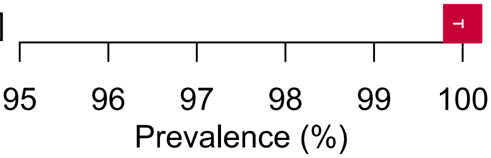

T

Study ID	Cases	Total	Prevalence	95% C.I.
**Spain (Specificity)**				
Agulló 2020	519	527	98.5	[97.4; 99.5]
Albert 2020	358	358	100.0	[99.6; 100.0]
Alemany 2020	450	455	98.9	[97.9; 99.9]
Linares 2020	133	133	100.0	[99.0; 100.0]
Torress 2021	555	555	100.0	[99.8; 100.0]
**Random effects model**		**2028**	**99.7**	**[99.2; 100.0]**

Heterogeneity: I^2 = 67%, τ^2 < 0.0001, χ^2_4 = 12.18 (p = 0.02)

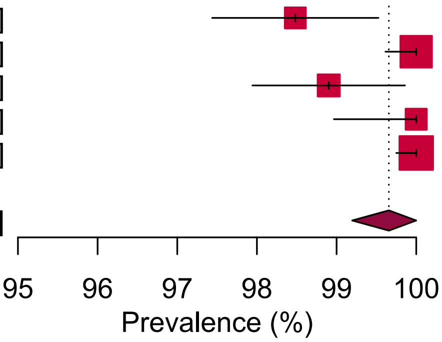

U

Study ID	Cases	Total	Prevalence	95% C.I.
**Thailand (Specificity)**				
Chaimayo 2020	389	394	98.7	[97.6; 99.8]

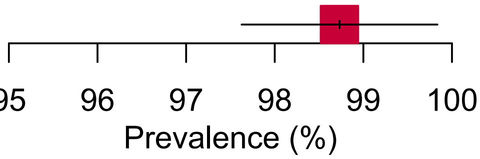

V

Study ID	Cases	Total	Prevalence	95% C.I.
**Uganda (Specificity)**				
Nalumansi 2020	159	172	92.4	[88.5; 96.4]

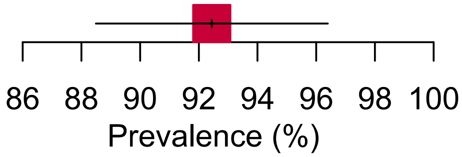

W

Study ID	Cases	Total	Prevalence	95% C.I.
**USA (Specificity)**				
Pilarowski 2020a	845	845	100.0	[99.8; 100]
Pilarowski 2020b	3062	3065	99.9	[99.8; 100]
**Random effects model**		**3910**	**99.9**	**[99.8; 100]**

Heterogeneity: $I^2 = 0\%$, $\tau^2 = 0$, $\chi^2_1 = 0.94$ ($p = 0.33$)

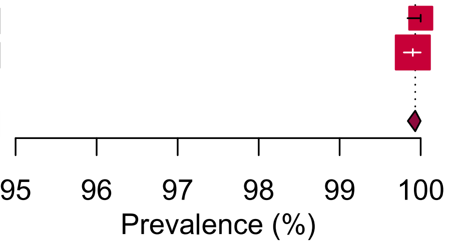

X

Study ID	Cases	Total	Prevalence	95% C.I.
**Abbott BinaxNOW™ (Specificity)**				
Pilarowski 2020a	845	845	100.0	[99.8; 100]
Pilarowski 2020b	3062	3065	99.9	[99.8; 100]
**Random effects model**		**3910**	**99.9**	**[99.8; 100]**

Heterogeneity: $I^2 = 0\%$, $\tau^2 = 0$, $\chi^2_1 = 0.94$ ($p = 0.33$)

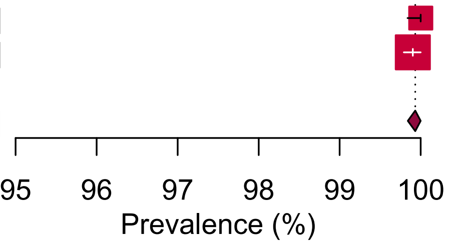

Y

Study ID	Cases	Total	Prevalence	95% C.I.
**Biocredit (Specificity)**				
Weitzel 2020	101	102	99.0	[97.1; 100]

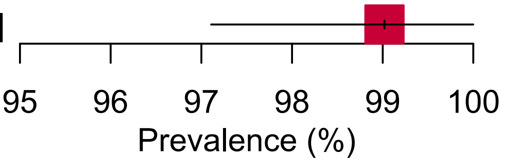

Z

Study ID	Cases	Total	Prevalence	95% C.I.
**Panbio™ (Specificity)**				
Agulló 2020	519	527	98.5	[97.4; 99.5]
Albert 2020	358	358	100.0	[99.6; 100.0]
Alemany 2020	450	455	98.9	[97.9; 99.9]
Fenollar 2020	130	137	94.9	[91.2; 98.6]
Gremmels 2020	1373	1373	100.0	[99.9; 100.0]
Linares 2020	133	133	100.0	[99.0; 100.0]
Strömer 2021	6	9	66.7	[35.9; 97.5]
Torress 2021	555	555	100.0	[99.8; 100.0]
**Random effects model**		**3547**	**99.7**	**[99.4; 100.0]**

Heterogeneity: $I^2 = 72\%$, $\tau^2 < 0.0001$, $\chi^2_7 = 24.78$ ($p < 0.01$)

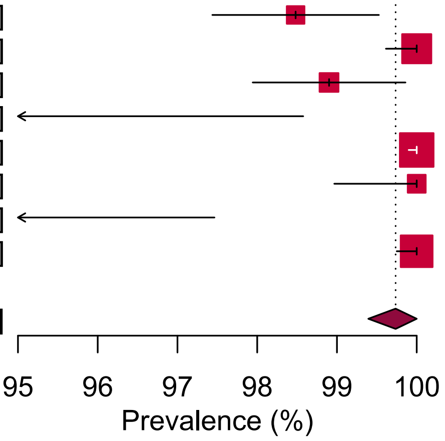

AA

Study ID	Cases	Total	Prevalence	95% C.I.
**Standard™ (Specificity)**				
Cerutti 2020	221	221	100.0	[99.4; 100.0]
Chaimayo 2020	389	394	98.7	[97.6; 99.8]
Gupta 2020	252	253	99.6	[98.8; 100.0]
Lindner 2020	249	250	99.6	[98.8; 100.0]
Liotti 2020	251	255	98.4	[96.9; 100.0]
Nalumansi 2020	159	172	92.4	[88.5; 96.4]
Torress 2021	555	555	100.0	[99.8; 100.0]
**Random effects model**		**2100**	**99.4**	**[98.8; 100.0]**

Heterogeneity: $I^2 = 74\%$, $\tau^2 < 0.0001$, $\chi^2_6 = 23.17$ ($p < 0.01$)

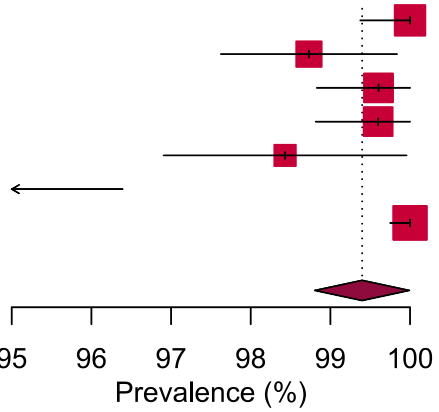

AB

Study ID	Cases	Total	Prevalence	95% C.I.
**Symptomatic (Sensitivity)**				
Agulló 2020	131	278	47.1	[41.3; 53.0]
Azzi 2020	25	27	92.6	[82.7; 100.0]
Fenollar 2020	144	182	79.1	[73.2; 85.0]
Gupta 2020	54	64	84.4	[75.5; 93.3]
Turcato 2020	152	169	89.9	[85.4; 94.5]
**Random effects model**		**720**	**78.5**	**[61.2; 95.9]**

Heterogeneity: $I^2 = 97\%$, $\tau^2 = 0.0378$, $\chi^2_4 = 143.42$ ($p < 0.01$)

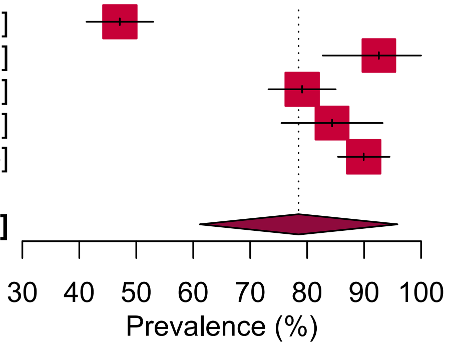

AC

Study ID	Cases	Total	Prevalence	95% C.I.
**Asymptomatic (Sensitivity)**				
Agulló 2020	20	100	20.0	[12.2; 27.8]
Azzi 2020	25	28	89.3	[77.8; 100.0]
Fenollar 2020	10	22	45.5	[24.6; 66.3]
Gupta 2020	9	13	69.2	[44.1; 94.3]
Turcato 2020	27	54	50.0	[36.7; 63.3]
**Random effects model**		**217**	**54.5**	**[24.3; 84.7]**

Heterogeneity: $I^2 = 96\%$, $\tau^2 = 0.1114$, $\chi^2_4 = 100.59$ ($p < 0.01$)

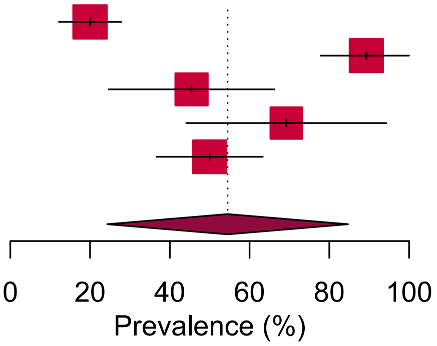

AD

Study ID	Cases	Total	Prevalence	95% C.I.
**Onset of symptoms < 5 days (Sensitivity)**				
Gupta 2020	58	70	82.9	[74.0; 91.7]
Linares 2020	29	34	85.3	[73.4; 97.2]
Lindner 2020	26	30	86.7	[74.5; 98.8]
Turcato 2020	179	223	80.3	[75.0; 85.5]
**Random effects model**		**357**	**82.0**	**[78.1; 86.0]**

Heterogeneity: $I^2 = 0\%$, $\tau^2 = 0$, $\chi^2_3 = 1.32$ ($p = 0.73$)

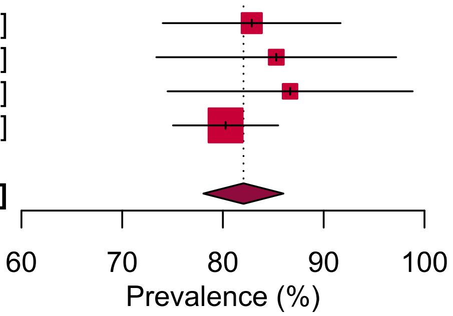

AE

Study ID	Cases	Total	Prevalence	95% C.I.
**Onset of symptoms > 5 days (Sensitivity)**				
Gupta 2020	5	7	71.4	[38.0; 100.0]
Linares 2020	39	50	78.0	[66.5; 89.5]
Lindner 2020	5	9	55.6	[23.1; 88.0]
**Random effects model**		**66**	**75.1**	**[64.8; 85.4]**

Heterogeneity: $I^2 = 0\%$, $\tau^2 = 0$, $\chi^2_2 = 1.68$ ($p = 0.43$)

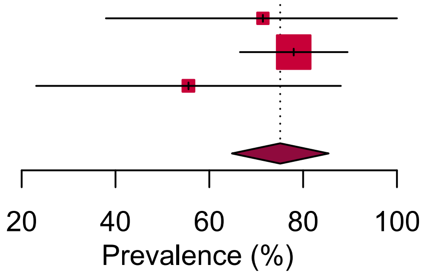

AF

Study ID	Cases	Total	Prevalence	95% C.I.
**Nasopharyngeal Swab (Sensitivity)**				
Agulló 2020	76	132	57.6	[49.1; 66.0]
Azzi 2020	26	28	92.9	[83.3; 100.0]
Lindner 2020	31	39	79.5	[66.8; 92.2]
Mak 2020a	55	105	52.4	[42.8; 61.9]
Mak 2020b	24	35	68.6	[53.2; 84.0]
**Random effects model**		**339**	**70.1**	**[54.1; 86.1]**

Heterogeneity: $I^2 = 91\%$, $\tau^2 = 0.0300$, $\chi^2_4 = 45.32$ ($p < 0.01$)

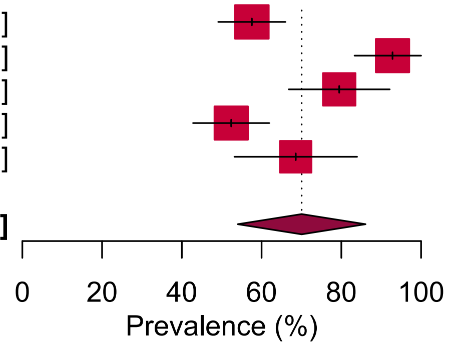

AG

Study ID	Cases	Total	Prevalence	95% C.I.
**Saliva (Sensitivity)**				
Agulló 2020	28	121	23.1	[15.6; 30.7]
Azzi 2020	50	55	90.9	[83.3; 98.5]
Yamayoshi 2020	40	108	37.0	[27.9; 46.1]
**Random effects model**		**284**	**50.4**	**[7.9; 92.9]**

Heterogeneity: $I^2 = 99\%$, $\tau^2 = 0.1393$, $\chi^2_2 = 167.76$ ($p < 0.01$)

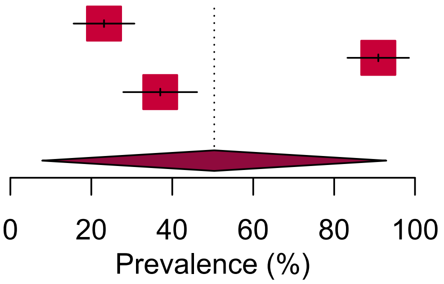

AH

Study ID	Cases	Total	Prevalence	95% C.I.
**Throat saliva or Swab (Sensitivity)**				
Mak 2020a	47	105	44.8	[35.3; 54.3]
Mak 2020b	24	35	68.6	[53.2; 84.0]
Mak 2020c	18	45	40.0	[25.7; 54.3]
Yamayoshi 2020	0	8	0.0	[0.0; 15.0]
**Random effects model**		**193**	**38.4**	**[13.7; 63.1]**

Heterogeneity: $I^2 = 93\%$, $\tau^2 = 0.0586$, $\chi^2_3 = 41.66$ ($p < 0.01$)

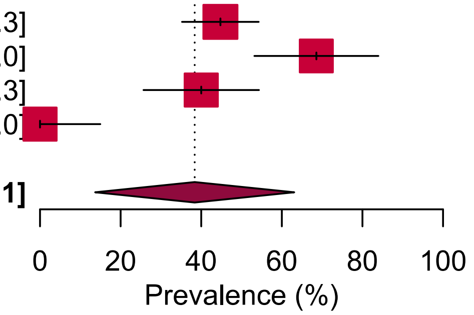

AI

Study ID	Cases	Total	Prevalence	95% C.I.
**Ct value ≤20 (Sensitivity)**				
Fenollar 2020	58	59	98.3	[95.0; 100]
Krüttgen 2020	5	5	100.0	[77.9; 100]
Lindner 2020	18	18	100.0	[92.8; 100]
Stromer 2020	10	10	100.0	[87.7; 100]
Yamayoshi 2020	16	16	100.0	[92.0; 100]
**Random effects model**		**108**	**98.8**	**[96.1; 100]**

Heterogeneity: I^2 = 0%, τ^2 = 0, χ^2_4 = 0.33 (p = 0.99)

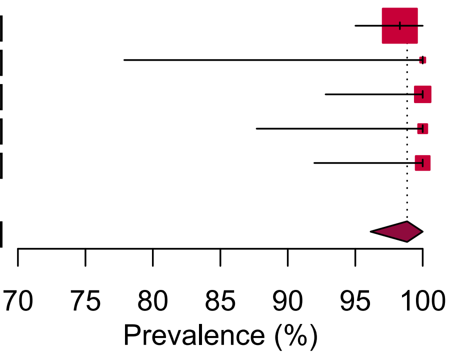

AJ

Study ID	Cases	Total	Prevalence	95% C.I.
**Ct value 21-25 (Sensitivity)**				
Fenollar 2020	49	52	94.2	[87.9; 100.0]
Krüttgen 2020	12	12	100.0	[89.5; 100.0]
Linares 2020	33	34	97.1	[91.4; 100.0]
Lindner 2020	7	8	87.5	[64.6; 100.0]
Stromer 2020	36	40	90.0	[80.7; 99.3]
Yamayoshi 2020	64	96	66.7	[57.2; 76.1]
**Random effects model**		**242**	**89.6**	**[80.1; 99.0]**

Heterogeneity: I^2 = 85%, τ^2 = 0.0110, χ^2_5 = 33.78 (p < 0.01)

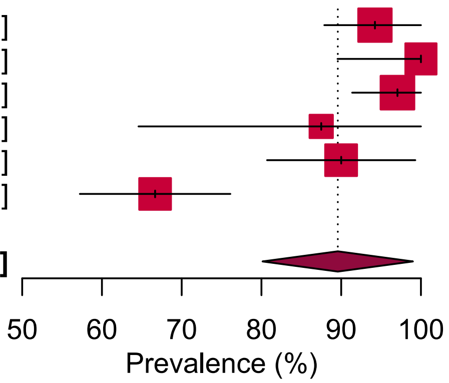

AK

Study ID	Cases	Total	Prevalence	95% C.I.
**Ct value 26-30 (Sensitivity)**				
Agulló 2020	5	28	17.9	[3.7; 32.0]
Fenollar 2020	39	53	73.6	[61.7; 85.5]
Krüttgen 2020	19	20	95.0	[85.4; 100.0]
Linares 2020	7	9	77.8	[50.6; 100.0]
Lindner 2020	4	7	57.1	[20.5; 93.8]
Stromer 2020	33	58	56.9	[44.2; 69.6]
Yamayoshi 2020	17	148	11.5	[6.3; 16.6]
**Random effects model**		**323**	**55.4**	**[24.0; 86.7]**

Heterogeneity: I^2 = 98%, τ^2 = 0.1695, χ^2_6 = 299.03 (p < 0.01)

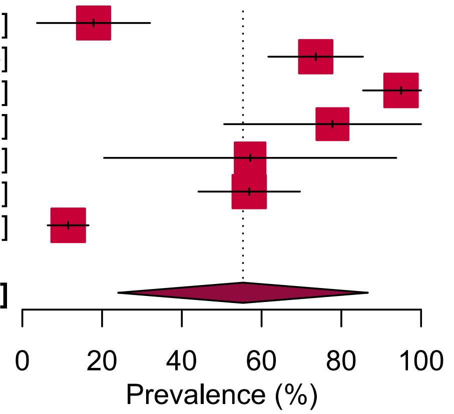

AL

Study ID	Cases	Total	Prevalence	95% C.I.
**Ct value 31-35 (Sensitivity)**				
Agulló 2020	8	58	13.8	[4.9; 22.7]
Fenollar 2020	8	40	20.0	[7.6; 32.4]
Krüttgen 2020	13	29	44.8	[26.7; 62.9]
Linares 2020	3	10	30.0	[1.6; 58.4]
Lindner 2020	1	6	16.7	[0.0; 46.5]
Stromer 2020	0	15	0.0	[0.0; 8.5]
Yamayoshi 2020	0	44	0.0	[0.0; 3.1]
**Random effects model**		**202**	**15.1**	**[4.5; 25.7]**

Heterogeneity: $I^2 = 86\%$, $\tau^2 = 0.0146$, $\chi^2_6 = 41.76$ ($p < 0.01$)

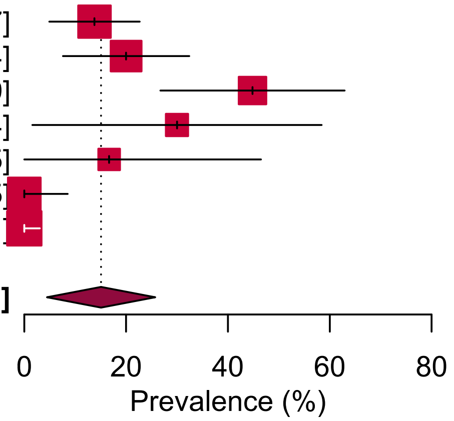

AM

Study ID	Cases	Total	Prevalence	95% C.I.
**Ct value 36-40 (Sensitivity)**				
Krüttgen 2020	2	9	22.2	[0; 49.4]
Linares 2020	1	7	14.3	[0; 40.2]
Stromer 2020	0	1	0.0	[0; 60.0]
**Random effects model**		**17**	**16.5**	**[0; 34.4]**

Heterogeneity: $I^2 = 0\%$, $\tau^2 = 0$, $\chi^2_2 = 0.49$ ($p = 0.78$)

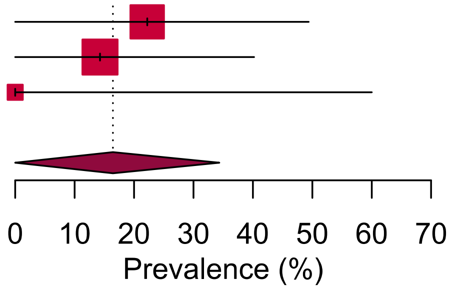

AN

Study ID	Cases	Total	Prevalence	95% C.I.
**Africa (Sensitivity)**				
Abdelrazik 2020	81	188	43.1	[36.0; 50.2]
Nalumansi 2020	63	90	70.0	[60.5; 79.5]
**Random effects model**		**278**	**56.4**	**[30.0; 82.7]**

Heterogeneity: $I^2 = 95\%$, $\tau^2 = 0.0344$, $\chi^2_1 = 19.91$ ($p < 0.01$)

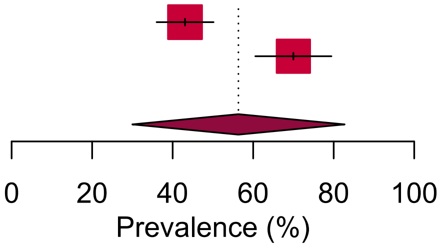

AO

Study ID	Cases	Total	Prevalence	95% C.I.
**Asia (Sensitivity)**				
Chaimayo 2020	59	60	98.3	[95.1; 100.0]
Diao 2020	152	201	75.6	[69.7; 81.6]
Gupta 2020	63	77	81.8	[73.2; 90.4]
Mak 2020a	94	140	67.1	[59.4; 74.9]
Mak 2020b	72	105	68.6	[59.7; 77.5]
Mak 2020c	51	160	31.9	[24.7; 39.1]
Yamayoshi 2020	97	304	31.9	[26.7; 37.1]
**Random effects model**		**1047**	**65.0**	**[42.3; 87.8]**

Heterogeneity: I^2 = 99%, τ^2 = 0.0931, χ^2_6 = 603.25 (p < 0.01)

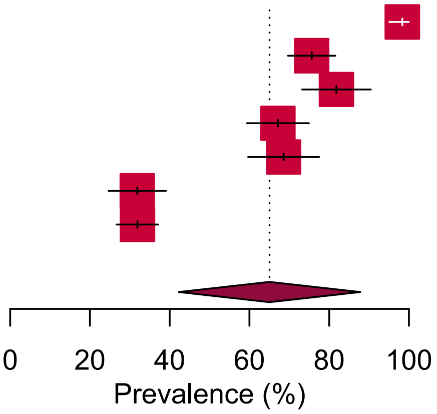

AP

Study ID	Cases	Total	Prevalence	95% C.I.
**Europe (Sensitivity)**				
Agulló 2020	76	132	57.6	[49.1; 66.0]
Albert 2020	43	54	79.6	[68.9; 90.4]
Alemany 2020	872	951	91.7	[89.9; 93.4]
Azzi 2020	76	83	91.6	[85.6; 97.5]
Cerutti 2020	77	109	70.6	[62.1; 79.2]
Fenollar 2020	154	204	75.5	[69.6; 81.4]
Gremmels 2020	152	202	75.2	[69.3; 81.2]
Krüttgen 2020	53	75	70.7	[60.4; 81.0]
Linares 2020	39	50	78.0	[66.5; 89.5]
Lindner 2020	31	39	79.5	[66.8; 92.2]
Liotti 2020	49	104	47.1	[37.5; 56.7]
Scohy 2020	32	106	30.2	[21.4; 38.9]
Strömer 2021	79	124	63.7	[55.2; 72.2]
Toptan 2021	45	58	77.6	[66.9; 88.3]
Torress 2021	38	79	48.1	[37.1; 59.1]
Turcato 2020	179	223	80.3	[75.0; 85.5]
**Random effects model**		**2593**	**70.0**	**[61.3; 78.6]**

Heterogeneity: I^2 = 96%, τ^2 = 0.0289, χ^2_{15} = 419.30 (p < 0.01)

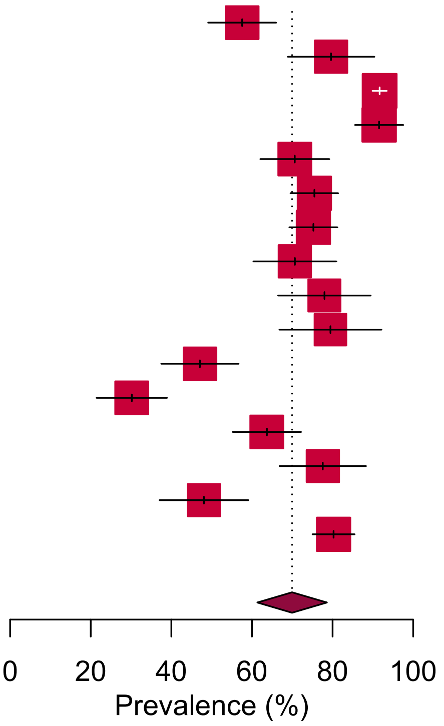

AQ

Study ID	Cases	Total	Prevalence	95% C.I.
**North America (Sensitivity)**				
Pilarowski 2020a	15	26	57.7	[38.7; 76.7]
Pilarowski 2020b	211	237	89.0	[85.1; 93.0]
**Random effects model**		**263**	**74.8**	**[44.2; 100.0]**

Heterogeneity: $I^2 = 90\%$, $\tau^2 = 0.0442$, $\chi^2_1 = 10.02$ ($p < 0.01$)

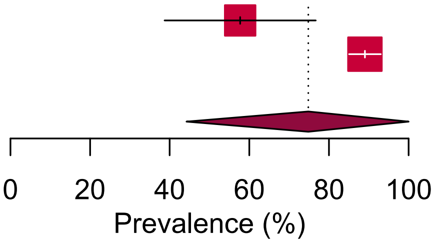

AR

Study ID	Cases	Total	Prevalence	95% C.I.
**South America (Sensitivity)**				
Porte 2020	77	82	93.9	[88.7; 99.1]
Weitzel 2020	130	246	52.8	[46.6; 59.1]
**Random effects model**		**328**	**73.4**	**[33.2; 100.0]**

Heterogeneity: $I^2 = 99\%$, $\tau^2 = 0.0834$, $\chi^2_1 = 98.51$ ($p < 0.01$)

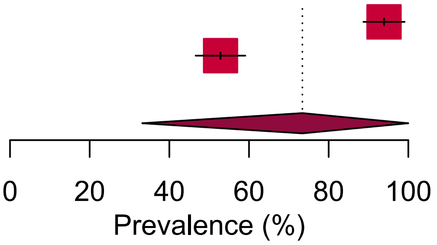

AS

Study ID	Cases	Total	Prevalence	95% C.I.
**Belgium (Sensitivity)**				
Scohy 2020	32	106	30.2	[21.4; 38.9]

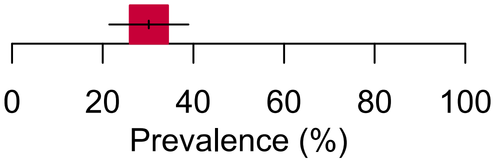

AT

Study ID	Cases	Total	Prevalence	95% C.I.
**Chile (Sensitivity)**				
Porte 2020	77	82	93.9	[88.7; 99.1]
Weitzel 2020	130	246	52.8	[46.6; 59.1]
**Random effects model**		**328**	**73.4**	**[33.2; 100.0]**

Heterogeneity: $I^2 = 99\%$, $\tau^2 = 0.0834$, $\chi^2_1 = 98.51$ ($p < 0.01$)

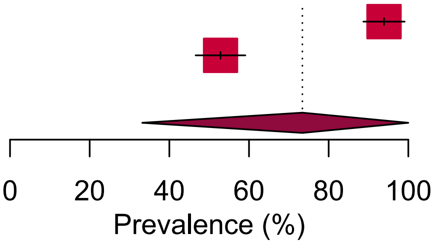

AU

Study ID	Cases	Total	Prevalence	95% C.I.
**China (Sensitivity)**				
Diao 2020	152	201	75.6	[69.7; 81.6]

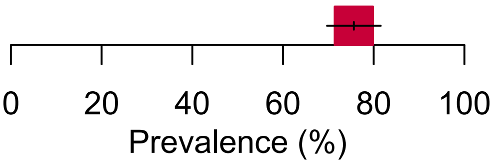

AV

Study ID	Cases	Total	Prevalence	95% C.I.
**France (Sensitivity)**				
Fenollar 2020	154	204	75.5	[69.6; 81.4]

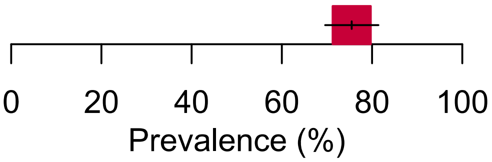

AW

Study ID	Cases	Total	Prevalence	95% C.I.
**Germany (Sensitivity)**				
Azzi 2020	76	83	91.6	[85.6; 97.5]
Cerutti 2020	77	109	70.6	[62.1; 79.2]
Krüttgen 2020	53	75	70.7	[60.4; 81.0]
Lindner 2020	31	39	79.5	[66.8; 92.2]
Liotti 2020	49	104	47.1	[37.5; 56.7]
Strömer 2021	79	124	63.7	[55.2; 72.2]
Toptan 2021	45	58	77.6	[66.9; 88.3]
Turcato 2020	179	223	80.3	[75.0; 85.5]
**Random effects model**		**815**	**72.8**	**[63.3; 82.3]**

Heterogeneity: $I^2 = 91\%$, $\tau^2 = 0.0167$, $\chi^2_7 = 74.44$ ($p < 0.01$)

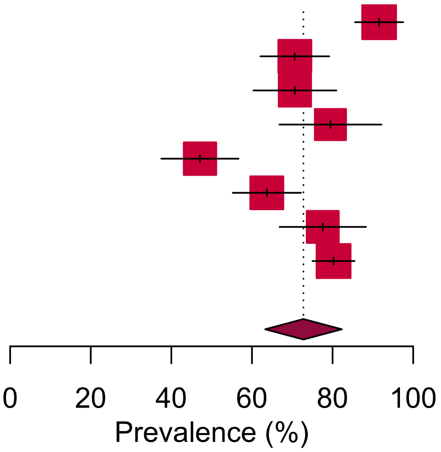

AX

Study ID	Cases	Total	Prevalence	95% C.I.
**Hong Kong (Sensitivity)**				
Mak 2020a	94	140	67.1	[59.4; 74.9]
Mak 2020b	72	105	68.6	[59.7; 77.5]
Mak 2020c	51	160	31.9	[24.7; 39.1]
**Random effects model**		**405**	**55.8**	**[31.3; 80.3]**

Heterogeneity: $I^2 = 97\%$, $\tau^2 = 0.0453$, $\chi^2_2 = 57.34$ ($p < 0.01$)

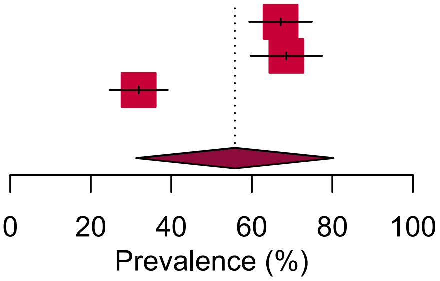

AY

Study ID	Cases	Total	Prevalence	95% C.I.
**India (Sensitivity)**				
Gupta 2020	63	77	81.8	[73.2; 90.4]

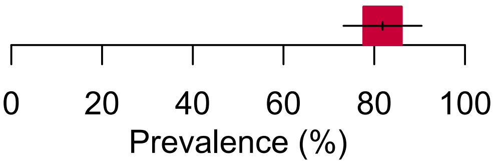

AZ

Study ID	Cases	Total	Prevalence	95% C.I.
**Italy (Sensitivity)**				
Azzi 2020	76	83	91.6	[85.6; 97.5]
Cerutti 2020	77	109	70.6	[62.1; 79.2]
Liotti 2020	49	104	47.1	[37.5; 56.7]
Turcato 2020	179	223	80.3	[75.0; 85.5]
**Random effects model**		**519**	**72.8**	**[56.7; 88.8]**

Heterogeneity: $I^2 = 95\%$, $\tau^2 = 0.0253$, $\chi^2_3 = 63.13$ ($p < 0.01$)

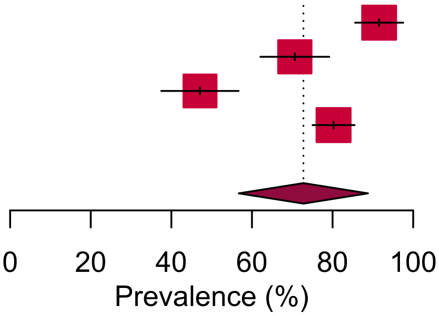

BA

Study ID	Cases	Total	Prevalence	95% C.I.
**Japan (Sensitivity)**				
Yamayoshi 2020	97	304	31.9	[26.7; 37.1]

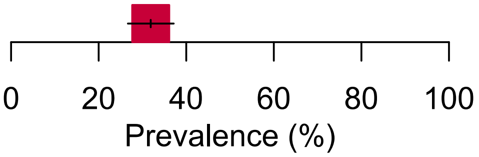

BB

Study ID	Cases	Total	Prevalence	95% C.I.
**Netherlands (Sensitivity)**				
Gremmels 2020	152	202	75.2	[69.3; 81.2]

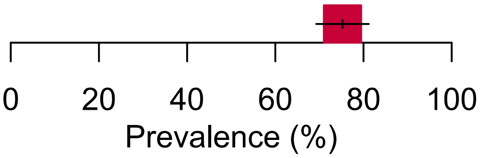

BC

Study ID	Cases	Total	Prevalence	95% C.I.
**Spain (Sensitivity)**				
Agulló 2020	76	132	57.6	[49.1; 66.0]
Albert 2020	43	54	79.6	[68.9; 90.4]
Alemany 2020	872	951	91.7	[89.9; 93.4]
Linares 2020	39	50	78.0	[66.5; 89.5]
Torress 2021	38	79	48.1	[37.1; 59.1]
**Random effects model**		**1266**	**71.2**	**[52.6; 89.8]**

Heterogeneity: I^2 = 97%, τ^2 = 0.0426, χ^2_4 = 121.75 (p < 0.01)

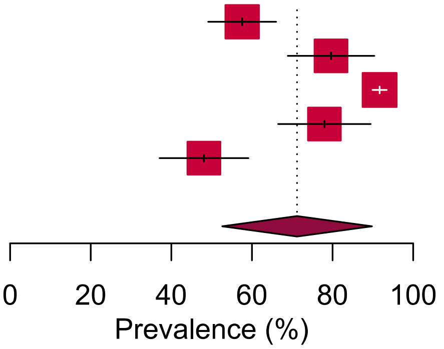

BD

Study ID	Cases	Total	Prevalence	95% C.I.
**Thailand (Sensitivity)**				
Chaimayo 2020	59	60	98.3	[95.1; 100]

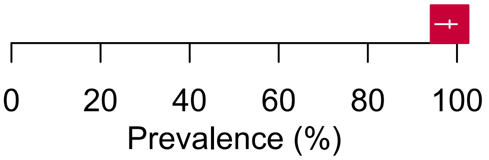

BE

Study ID	Cases	Total	Prevalence	95% C.I.
**Uganda (Sensitivity)**				
Nalumansi 2020	63	90	70.0	[60.5; 79.5]

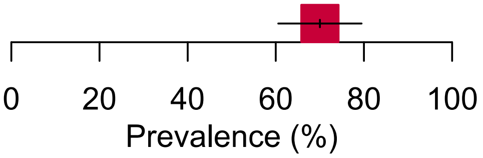

BF

Study ID	Cases	Total	Prevalence	95% C.I.
**USA (Sensitivity)**				
Pilarowski 2020a	15	26	57.7	[38.7; 76.7]
Pilarowski 2020b	211	237	89.0	[85.1; 93.0]
**Random effects model**		**263**	**74.8**	**[44.2; 100.0]**

Heterogeneity: I^2 = 90%, τ^2 = 0.0442, χ^2_1 = 10.02 (p < 0.01)

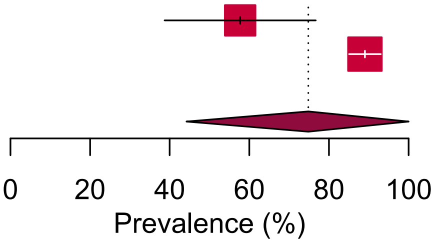

BG

Study ID	Cases	Total	Prevalence	95% C.I.
**Abbott BinaxNOW™ (Sensitivity)**				
Pilarowski 2020a	15	26	57.7	[38.7; 76.7]
Pilarowski 2020b	211	237	89.0	[85.1; 93.0]
**Random effects model**		**263**	**74.8**	**[44.2; 100.0]**

Heterogeneity: I^2 = 90%, τ^2 = 0.0442, χ^2_1 = 10.02 (p < 0.01)

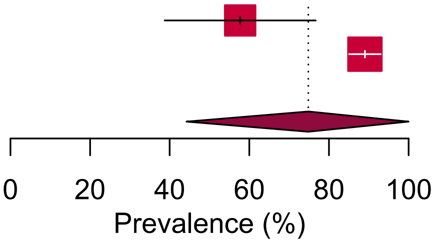

BH

Study ID	Cases	Total	Prevalence	95% C.I.
**Biocredit (Sensitivity)**				
Abdelrazik 2020	81	188	43.1	[36.0; 50.2]
Mak 2020c	51	160	31.9	[24.7; 39.1]
Weitzel 2020	130	246	52.8	[46.6; 59.1]
**Random effects model**		**594**	**42.7**	**[30.7; 54.7]**

Heterogeneity: I^2 = 89%, τ^2 = 0.0101, χ^2_2 = 18.59 (p < 0.01)

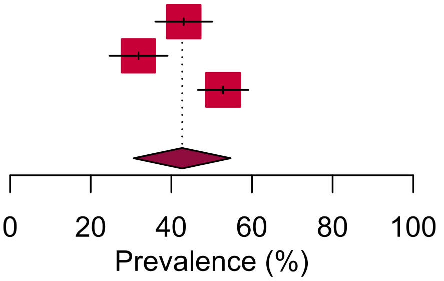

BI

Study ID	Cases	Total	Prevalence	95% C.I.
**Panbio™ (Sensitivity)**				
Agulló 2020	76	132	57.6	[49.1; 66.0]
Albert 2020	43	54	79.6	[68.9; 90.4]
Alemany 2020	872	951	91.7	[89.9; 93.4]
Fenollar 2020	154	204	75.5	[69.6; 81.4]
Gremmels 2020	152	202	75.2	[69.3; 81.2]
Linares 2020	39	50	78.0	[66.5; 89.5]
Mak 2020b	72	105	68.6	[59.7; 77.5]
Strömer 2021	12	12	100.0	[89.5; 100.0]
Torress 2021	38	79	48.1	[37.1; 59.1]
**Random effects model**		**1789**	**75.1**	**[64.9; 85.3]**

Heterogeneity: I^2 = 96%, τ^2 = 0.0223, χ^2_8 = 178.29 (p < 0.01)

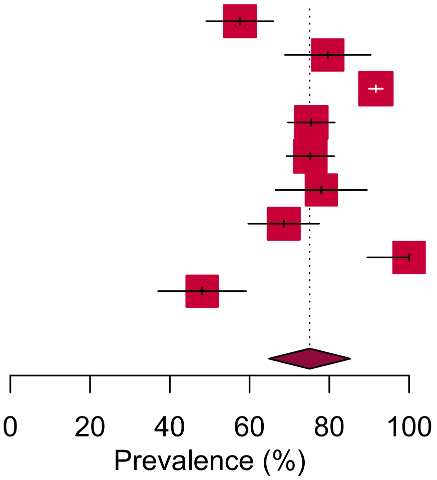

BJ

Study ID	Cases	Total	Prevalence	95% C.I.
**Standard™ (Sensitivity)**				
Cerutti 2020	77	109	70.6	[62.1; 79.2]
Chaimayo 2020	59	60	98.3	[95.1; 100.0]
Gupta 2020	63	77	81.8	[73.2; 90.4]
Lindner 2020	31	39	79.5	[66.8; 92.2]
Liotti 2020	49	104	47.1	[37.5; 56.7]
Nalumansi 2020	63	90	70.0	[60.5; 79.5]
Torress 2021	38	79	48.1	[37.1; 59.1]
Yamayoshi 2020	26	76	34.2	[23.5; 44.9]
**Random effects model**		**634**	**66.4**	**[48.5; 84.2]**

Heterogeneity: $I^2 = 97\%$, $\tau^2 = 0.0639$, $\chi^2_7 = 274.38$ ($p < 0.01$)

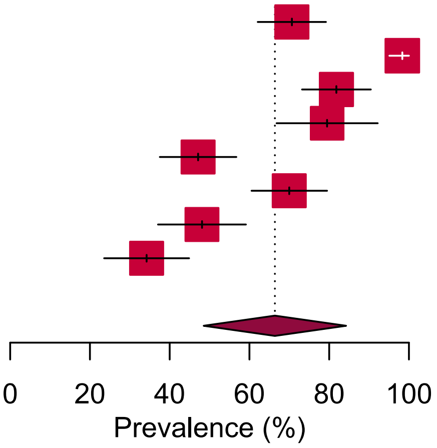

Figure S1. Subgroup analyses based on the presence of symptoms, symptom onset, specimen types, Ct values, continents, countries, and kit manufacturers estimating the pooled specificity (A-AA) and sensitivity (AB-BJ) using rapid antigen test kit to diagnose SARS-CoV-2.
