# Supplementary material for: Diagnostic Accuracy of Rapid Antigen Test Kits for Detecting SARS-CoV-2: A Systematic Review and Meta-Analysis of 17,171 Suspected COVID-19 Patients
Source: J Clin Med. 2021 Aug 8;10(16):3493. doi: 10.3390/jcm10163493 (PMC8397079; doi:10.3390/jcm10163493)
Supplement: Supplementary file 1 [file jcm-10-03493-s001.zip › Supplementary Files/Figure S2_Sensitivity analyses.pdf]

A

Study ID	Cases	Total	Prevalence	95% C.I.
**Excluding small studies (n<100) (Specificity)**				
Agulló 2020	519	527	98.5	[97.4; 99.5]
Albert 2020	358	358	100.0	[99.6; 100.0]
Alemany 2020	450	455	98.9	[97.9; 99.9]
Azzi 2020	73	149	49.0	[41.0; 57.0]
Cerutti 2020	221	221	100.0	[99.4; 100.0]
Chaimayo 2020	389	394	98.7	[97.6; 99.8]
Fenollar 2020	130	137	94.9	[91.2; 98.6]
Gremmels 2020	1373	1373	100.0	[99.9; 100.0]
Gupta 2020	252	253	99.6	[98.8; 100.0]
Linares 2020	133	133	100.0	[99.0; 100.0]
Lindner 2020	249	250	99.6	[98.8; 100.0]
Liotti 2020	251	255	98.4	[96.9; 100.0]
Nalumansi 2020	159	172	92.4	[88.5; 96.4]
Pilarowski 2020a	845	845	100.0	[99.8; 100.0]
Pilarowski 2020b	3062	3065	99.9	[99.8; 100.0]
Torress 2021	555	555	100.0	[99.8; 100.0]
Turcato 2020	3157	3187	99.1	[98.7; 99.4]
Weitzel 2020	101	102	99.0	[97.1; 100.0]
**Random effects model**		**12431**	**99.4**	**[99.1; 99.8]**

Heterogeneity: $I^2 = 93\%$, $\tau^2 < 0.0001$, $\chi^2_{17} = 226.72$ ($p < 0.01$)

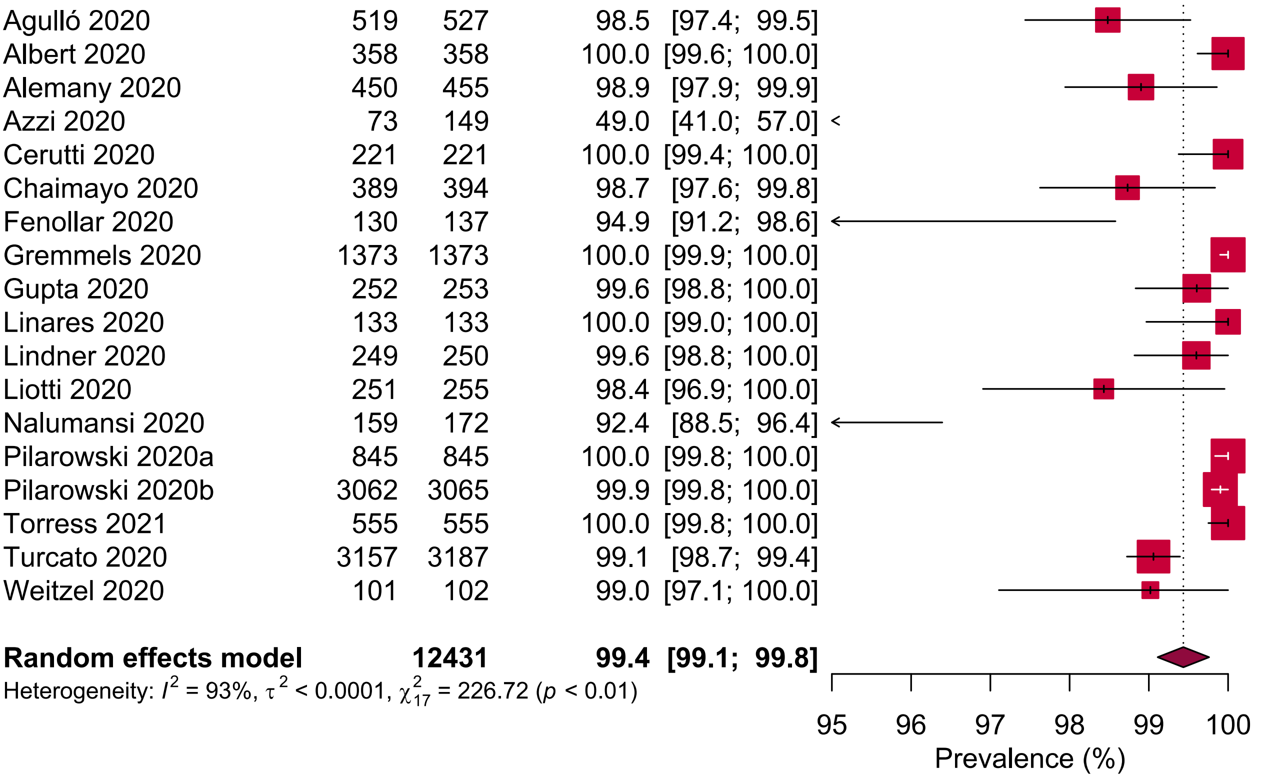

B

Study ID	Cases	Total	Prevalence	95% C.I.
**Excluding low- and medium-quality studies (Specificity)**				
Albert 2020	358	358	100.0	[99.6; 100.0]
Gremmels 2020	1373	1373	100.0	[99.9; 100.0]
Gupta 2020	252	253	99.6	[98.8; 100.0]
Liotti 2020	251	255	98.4	[96.9; 100.0]
Nalumansi 2020	159	172	92.4	[88.5; 96.4]
Porte 2020	45	45	100.0	[97.0; 100.0]
**Random effects model**		**2456**	**99.6**	**[99.1; 100.0]**

Heterogeneity: $I^2 = 74\%$, $\tau^2 < 0.0001$, $\chi^2_{5} = 19.01$ ($p < 0.01$)

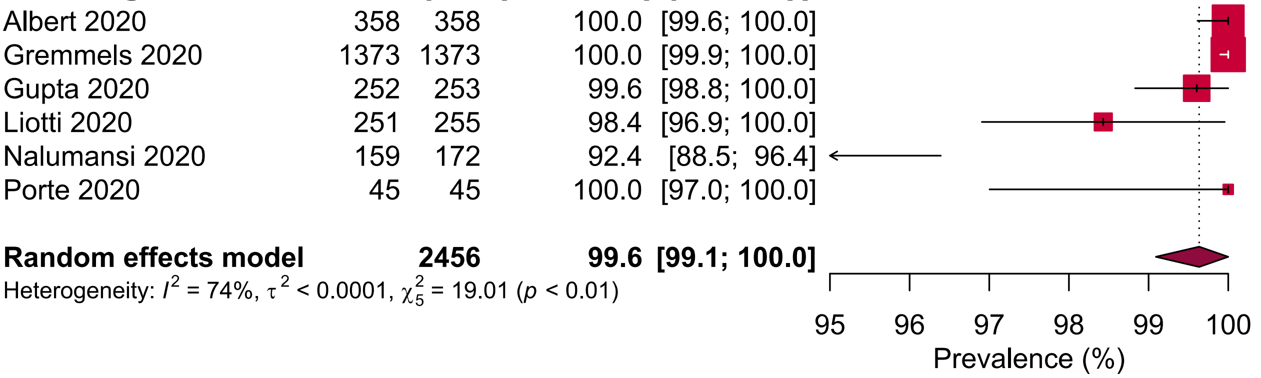

C

Study ID	Cases	Total	Prevalence	95% C.I.
**Using a fixed-effects model (Specificity)**				
Agulló 2020	519	527	98.5	[97.4; 99.5]
Albert 2020	358	358	100.0	[99.6; 100.0]
Alemany 2020	450	455	98.9	[97.9; 99.9]
Azzi 2020	73	149	49.0	[41.0; 57.0]
Cerutti 2020	221	221	100.0	[99.4; 100.0]
Chaimayo 2020	389	394	98.7	[97.6; 99.8]
Diao 2020	50	50	100.0	[97.3; 100.0]
Fenollar 2020	130	137	94.9	[91.2; 98.6]
Gremmels 2020	1373	1373	100.0	[99.9; 100.0]
Gupta 2020	252	253	99.6	[98.8; 100.0]
Krüttgen 2020	72	75	96.0	[91.6; 100.0]
Linares 2020	133	133	100.0	[99.0; 100.0]
Lindner 2020	249	250	99.6	[98.8; 100.0]
Liotti 2020	251	255	98.4	[96.9; 100.0]
Nalumansi 2020	159	172	92.4	[88.5; 96.4]
Pilarowski 2020a	845	845	100.0	[99.8; 100.0]
Pilarowski 2020b	3062	3065	99.9	[99.8; 100.0]
Porte 2020	45	45	100.0	[97.0; 100.0]
Scohy 2020	42	42	100.0	[96.8; 100.0]
Strömer 2021	10	10	100.0	[87.7; 100.0]
Toptan 2021	9	9	100.0	[86.5; 100.0]
Torress 2021	555	555	100.0	[99.8; 100.0]
Turcato 2020	3157	3187	99.1	[98.7; 99.4]
Weitzel 2020	101	102	99.0	[97.1; 100.0]
**Fixed effect model**		**12662**	**99.9**	**[99.8; 100.0]**
**Random effects model**			**99.4**	**[99.1; 99.8]**

Heterogeneity: $I^2 = 90\%$, $\tau^2 < 0.0001$, $\chi^2_{23} = 229.71$ ($p < 0.01$)

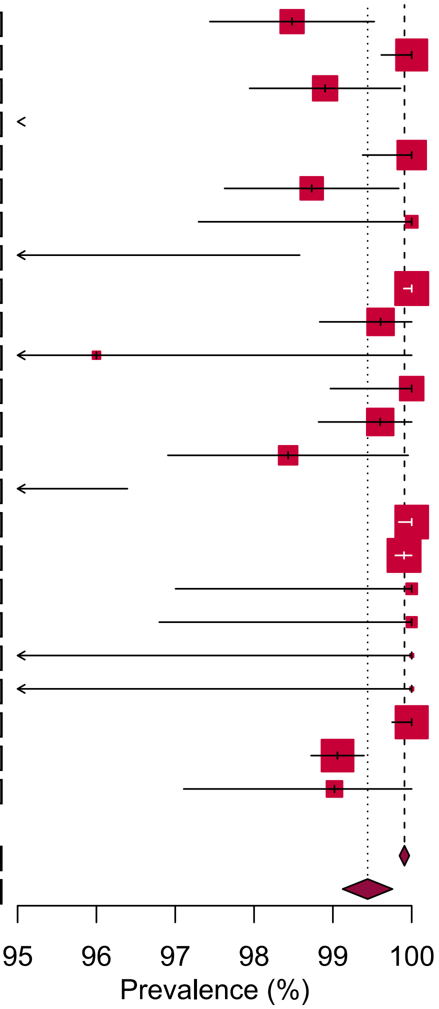

D

Study ID	Cases	Total	Prevalence	95% C.I.
**Excluding outlier studies (Specificity)**				
Agulló 2020	519	527	98.5	[97.4; 99.5]
Albert 2020	358	358	100.0	[99.6; 100.0]
Alemany 2020	450	455	98.9	[97.9; 99.9]
Cerutti 2020	221	221	100.0	[99.4; 100.0]
Chaimayo 2020	389	394	98.7	[97.6; 99.8]
Diao 2020	50	50	100.0	[97.3; 100.0]
Fenollar 2020	130	137	94.9	[91.2; 98.6]
Gremmels 2020	1373	1373	100.0	[99.9; 100.0]
Gupta 2020	252	253	99.6	[98.8; 100.0]
Krüttgen 2020	72	75	96.0	[91.6; 100.0]
Linares 2020	133	133	100.0	[99.0; 100.0]
Lindner 2020	249	250	99.6	[98.8; 100.0]
Liotti 2020	251	255	98.4	[96.9; 100.0]
Nalumansi 2020	159	172	92.4	[88.5; 96.4]
Pilarowski 2020a	845	845	100.0	[99.8; 100.0]
Pilarowski 2020b	3062	3065	99.9	[99.8; 100.0]
Porte 2020	45	45	100.0	[97.0; 100.0]
Scohy 2020	42	42	100.0	[96.8; 100.0]
Strömer 2021	10	10	100.0	[87.7; 100.0]
Toptan 2021	9	9	100.0	[86.5; 100.0]
Torress 2021	555	555	100.0	[99.8; 100.0]
Turcato 2020	3157	3187	99.1	[98.7; 99.4]
Weitzel 2020	101	102	99.0	[97.1; 100.0]
**Random effects model**		**12513**	**99.7**	**[99.5; 99.9]**

Heterogeneity: $I^2 = 71\%$, $\tau^2 < 0.0001$, $\chi^2_{22} = 75.14$ ($p < 0.01$)

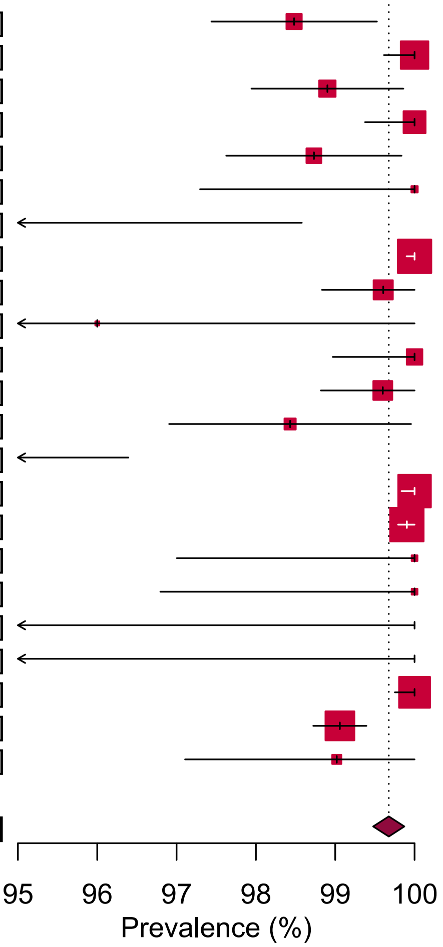

E

Study ID	Cases	Total	Prevalence	95% C.I.
**Excluding small studies (n<100) (Sensitivity)**				
Abdelrazik 2020	81	188	43.1	[36.0; 50.2]
Agulló 2020	76	132	57.6	[49.1; 66.0]
Alemany 2020	872	951	91.7	[89.9; 93.4]
Cerutti 2020	77	109	70.6	[62.1; 79.2]
Diao 2020	152	201	75.6	[69.7; 81.6]
Fenollar 2020	154	204	75.5	[69.6; 81.4]
Gremmels 2020	152	202	75.2	[69.3; 81.2]
Liotti 2020	49	104	47.1	[37.5; 56.7]
Mak 2020a	94	140	67.1	[59.4; 74.9]
Mak 2020b	72	105	68.6	[59.7; 77.5]
Mak 2020c	51	160	31.9	[24.7; 39.1]
Pilarowski 2020b	211	237	89.0	[85.1; 93.0]
Scohy 2020	32	106	30.2	[21.4; 38.9]
Strömer 2021	79	124	63.7	[55.2; 72.2]
Turcato 2020	179	223	80.3	[75.0; 85.5]
Weitzel 2020	130	246	52.8	[46.6; 59.1]
Yamayoshi 2020	97	304	31.9	[26.7; 37.1]
**Random effects model**		**3736**	**62.0**	**[51.1; 72.9]**

Heterogeneity: $I^2 = 99\%$, $\tau^2 = 0.0511$, $\chi^2_{16} = 1076.36$ ($p < 0.01$)

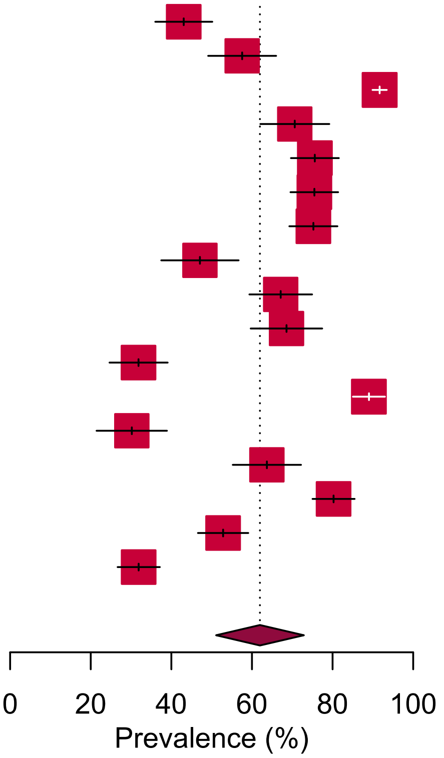

F

Study ID	Cases	Total	Prevalence	95% C.I.
**Excluding low- and medium-quality studies (Sensitivity)**				
Abdelrazik 2020	81	188	43.1	[36.0; 50.2]
Albert 2020	43	54	79.6	[68.9; 90.4]
Gremmels 2020	152	202	75.2	[69.3; 81.2]
Gupta 2020	63	77	81.8	[73.2; 90.4]
Liotti 2020	49	104	47.1	[37.5; 56.7]
Mak 2020a	94	140	67.1	[59.4; 74.9]
Nalumansi 2020	63	90	70.0	[60.5; 79.5]
Porte 2020	77	82	93.9	[88.7; 99.1]
**Random effects model**		**937**	**69.8**	**[56.7; 82.9]**

Heterogeneity: $I^2 = 96\%$, $\tau^2 = 0.0340$, $\chi^2_{7} = 167.74$ ($p < 0.01$)

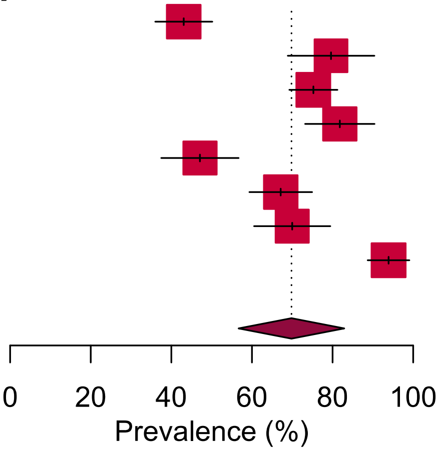

G

Study ID	Cases	Total	Prevalence	95% C.I.
**Using a fixed-effects model (Sensitivity)**				
Abdelrazik 2020	81	188	43.1	[36.0; 50.2]
Agulló 2020	76	132	57.6	[49.1; 66.0]
Albert 2020	43	54	79.6	[68.9; 90.4]
Alemany 2020	872	951	91.7	[89.9; 93.4]
Azzi 2020	76	83	91.6	[85.6; 97.5]
Cerutti 2020	77	109	70.6	[62.1; 79.2]
Chaimayo 2020	59	60	98.3	[95.1; 100.0]
Diao 2020	152	201	75.6	[69.7; 81.6]
Fenollar 2020	154	204	75.5	[69.6; 81.4]
Gremmels 2020	152	202	75.2	[69.3; 81.2]
Gupta 2020	63	77	81.8	[73.2; 90.4]
Krüttgen 2020	53	75	70.7	[60.4; 81.0]
Linares 2020	39	50	78.0	[66.5; 89.5]
Lindner 2020	31	39	79.5	[66.8; 92.2]
Liotti 2020	49	104	47.1	[37.5; 56.7]
Mak 2020a	94	140	67.1	[59.4; 74.9]
Mak 2020b	72	105	68.6	[59.7; 77.5]
Mak 2020c	51	160	31.9	[24.7; 39.1]
Nalumansi 2020	63	90	70.0	[60.5; 79.5]
Pilarowski 2020a	15	26	57.7	[38.7; 76.7]
Pilarowski 2020b	211	237	89.0	[85.1; 93.0]
Porte 2020	77	82	93.9	[88.7; 99.1]
Scohy 2020	32	106	30.2	[21.4; 38.9]
Strömer 2021	79	124	63.7	[55.2; 72.2]
Toptan 2021	45	58	77.6	[66.9; 88.3]
Torress 2021	38	79	48.1	[37.1; 59.1]
Turcato 2020	179	223	80.3	[75.0; 85.5]
Weitzel 2020	130	246	52.8	[46.6; 59.1]
Yamayoshi 2020	97	304	31.9	[26.7; 37.1]
**Fixed effect model**		**4509**	**79.9**	**[78.9; 80.9]**
**Random effects model**			**68.4**	**[60.8; 75.9]**

Heterogeneity: I^2 = 98%, τ^2 = 0.0415, χ^2_{28} = 1312.10 (p < 0.01)

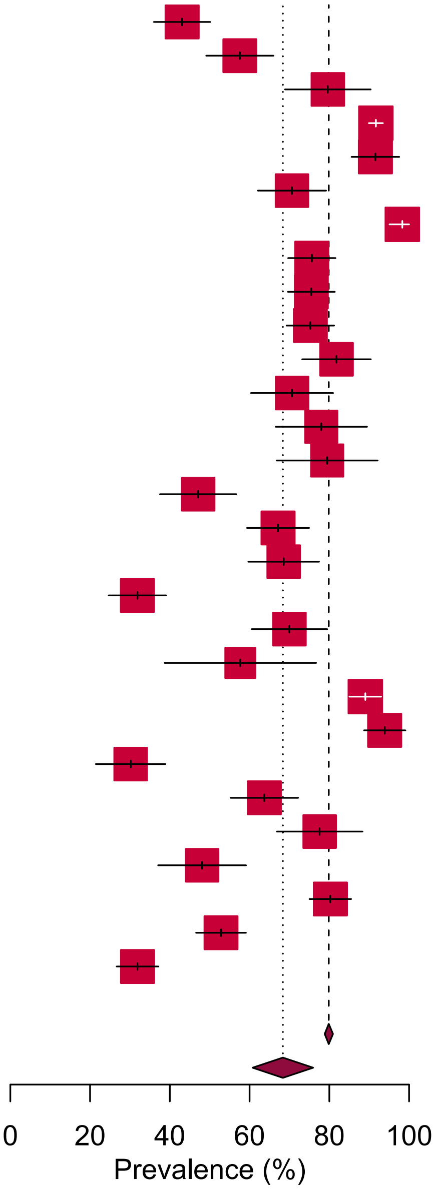

H

Study ID	Cases	Total	Prevalence	95% C.I.
**Excluding outlier studies (Sensitivity)**				
Abdelrazik 2020	81	188	43.1	[36.0; 50.2]
Agulló 2020	76	132	57.6	[49.1; 66.0]
Albert 2020	43	54	79.6	[68.9; 90.4]
Alemany 2020	872	951	91.7	[89.9; 93.4]
Azzi 2020	76	83	91.6	[85.6; 97.5]
Cerutti 2020	77	109	70.6	[62.1; 79.2]
Chaimayo 2020	59	60	98.3	[95.1; 100.0]
Diao 2020	152	201	75.6	[69.7; 81.6]
Fenollar 2020	154	204	75.5	[69.6; 81.4]
Gremmels 2020	152	202	75.2	[69.3; 81.2]
Gupta 2020	63	77	81.8	[73.2; 90.4]
Krüttgen 2020	53	75	70.7	[60.4; 81.0]
Linares 2020	39	50	78.0	[66.5; 89.5]
Lindner 2020	31	39	79.5	[66.8; 92.2]
Liotti 2020	49	104	47.1	[37.5; 56.7]
Mak 2020a	94	140	67.1	[59.4; 74.9]
Mak 2020b	72	105	68.6	[59.7; 77.5]
Nalumansi 2020	63	90	70.0	[60.5; 79.5]
Pilarowski 2020a	15	26	57.7	[38.7; 76.7]
Pilarowski 2020b	211	237	89.0	[85.1; 93.0]
Porte 2020	77	82	93.9	[88.7; 99.1]
Scohy 2020	32	106	30.2	[21.4; 38.9]
Strömer 2021	79	124	63.7	[55.2; 72.2]
Toptan 2021	45	58	77.6	[66.9; 88.3]
Torress 2021	38	79	48.1	[37.1; 59.1]
Turcato 2020	179	223	80.3	[75.0; 85.5]
Weitzel 2020	130	246	52.8	[46.6; 59.1]
**Random effects model**		**4045**	**71.2**	**[64.8; 77.6]**

Heterogeneity: $I^2 = 97\%$, $\tau^2 = 0.0267$, $\chi^2_{26} = 789.35$ ($p < 0.01$)

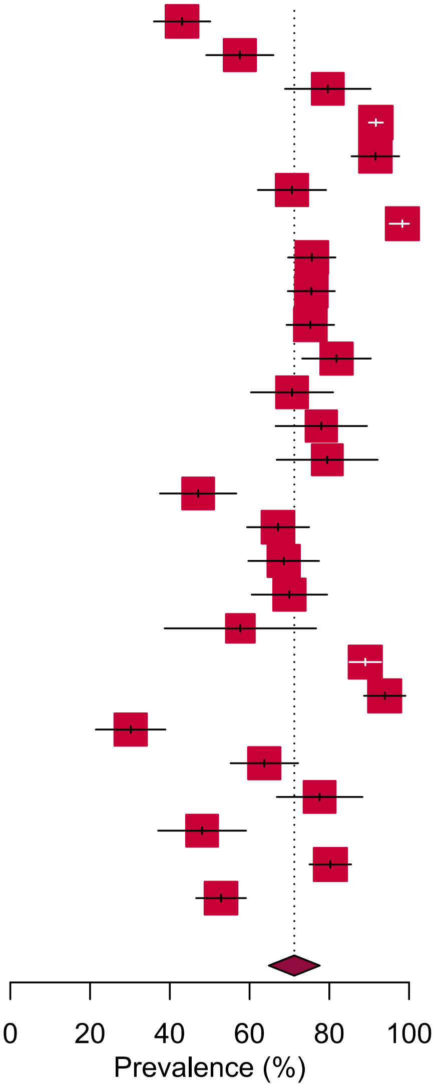

Figure S2. Sensitivity analyses excluding small studies (<100), excluding low- or medium-quality studies, using a fixed-effects model, and excluding outlier studies estimating polled specificity (A-D) and sensitivity (E-H) using rapid antigen test kit to diagnose SARS-CoV-2.
