# Supplementary material for: Diagnostic Accuracy of Rapid Antigen Test Kits for Detecting SARS-CoV-2: A Systematic Review and Meta-Analysis of 17,171 Suspected COVID-19 Patients
Source: J Clin Med. 2021 Aug 8;10(16):3493. doi: 10.3390/jcm10163493 (PMC8397079; doi:10.3390/jcm10163493)
Supplement: Supplementary file 1 [file jcm-10-03493-s001.zip › Supplementary Files/Table S1_Search strategy.pdf]

Table S1. Search strategies

Databases	Search strategies
**PubMed**	((rapid[Title]) AND (antigen[Title] OR detection[Title] OR diagnostic[Title] OR diagnosis[Title] OR test[Title] OR tests[Title] OR testing[Title] OR assay[Title] OR assays[Title])) AND (Coronavirus[Title] OR COVID-19[Title] OR COVID19[Title] OR nCoV[Title] OR SARS-CoV-2[Title] OR SARS-CoV2[Title])
**Scopus**	TITLE(rapid) AND TITLE(antigen OR detection OR diagnostic OR diagnosis OR test OR tests OR testing OR assay OR assays) AND TITLE(Coronavirus OR COVID-19 OR COVID19 OR nCoV OR SARS-CoV-2 OR SARS-CoV2)
**Web of Science**	TI=(rapid) AND TI=(antigen OR detection OR diagnostic OR diagnosis OR test OR tests OR testing OR assay OR assays) AND TI=(Coronavirus OR COVID-19 OR COVID19 OR nCoV OR SARS-CoV-2 OR SARS-CoV2)
**Google Scholar**	allintitle:(rapid) (antigen OR detection OR diagnostic OR diagnosis OR test OR tests OR testing OR assay OR assays) (Coronavirus OR COVID-19 OR COVID19 OR nCoV OR SARS-CoV-2 OR SARS-CoV2)
