# Supplementary material for: Diagnostic Accuracy of Rapid Antigen Test Kits for Detecting SARS-CoV-2: A Systematic Review and Meta-Analysis of 17,171 Suspected COVID-19 Patients
Source: J Clin Med. 2021 Aug 8;10(16):3493. doi: 10.3390/jcm10163493 (PMC8397079; doi:10.3390/jcm10163493)
Supplement: Supplementary file 1 [file jcm-10-03493-s001.zip › Supplementary Files/Table S2_Quality assessment.pdf]

**Table S2.** Quality assessment of the included studies

| Study ID | Quality assessment checklist for diagnostic accuracy | | | | | | | | | | % Yes |
|---|---|---|---|---|---|---|---|---|---|---|---|
| | 1 | 2 | 3 | 4 | 5 | 6 | 7 | 8 | 9 | 10 | |
| Abdelrazik 2020 | Y | Y | Y | N | NA | Y | N | Y | Y | Y | 77.7 |
| Agulló 2020 | Y | Y | Y | N | NA | Y | N | U | Y | Y | 66.6 |
| Albert 2020 | Y | Y | Y | N | NA | Y | N | Y | Y | Y | 77.7 |
| Alemany 2020 | Y | Y | Y | N | NA | Y | N | U | Y | Y | 66.6 |
| Azzi 2020 | Y | Y | U | Y | NA | Y | Y | U | Y | N | 66.6 |
| Cerutti 2020 | Y | Y | Y | N | NA | Y | N | U | Y | Y | 66.6 |
| Chaimayo 2020 | Y | Y | Y | N | NA | Y | N | U | Y | Y | 66.6 |
| Diao 2020 | Y | Y | U | Y | NA | Y | Y | U | Y | N | 66.6 |
| Fenollar 2020 | Y | Y | Y | N | NA | Y | N | U | Y | Y | 66.6 |
| Gremmels 2020 | Y | Y | Y | Y | NA | Y | Y | U | Y | Y | 88.8 |
| Gupta 2020 | Y | Y | Y | Y | NA | Y | Y | U | Y | Y | 88.8 |
| Krüttgen 2020 | Y | N | Y | N | NA | Y | N | U | Y | Y | 55.5 |
| Linares 2020 | Y | Y | Y | N | NA | Y | N | U | Y | Y | 66.6 |
| Lindner 2020 | Y | Y | U | Y | NA | Y | Y | U | Y | N | 66.6 |
| Liotti 2020 | Y | Y | Y | N | NA | Y | N | Y | Y | Y | 77.7 |
| Mak 2020a | Y | Y | Y | Y | NA | Y | Y | N | Y | Y | 88.8 |
| Mak 2020b | Y | Y | Y | N | NA | Y | N | U | Y | Y | 66.6 |
| Mak 2020c | Y | Y | Y | N | NA | Y | N | U | Y | U | 55.5 |
| Nalumansi 2020 | Y | Y | Y | Y | NA | Y | Y | U | Y | Y | 88.8 |
| Pilarowski 2020a | Y | Y | Y | N | NA | Y | N | U | Y | Y | 66.6 |
| Pilarowski 2020b | Y | Y | Y | N | NA | Y | N | U | Y | Y | 66.6 |
| Porte 2020 | Y | Y | Y | Y | NA | Y | Y | U | Y | Y | 88.8 |

| | | | | | | | | | | |
|---|---|---|---|---|---|---|---|---|---|---|
| Scohy 2020 | Y | Y | Y | N | NA | Y | N | U | Y | Y | 66.6 |
| Strömer 2021 | Y | Y | Y | N | NA | Y | N | U | Y | Y | 66.6 |
| Toptan 2021 | Y | Y | Y | U | NA | Y | U | U | Y | Y | 66.6 |
| Torress 2021 | Y | Y | Y | N | NA | Y | N | U | Y | Y | 66.6 |
| Turcato 2020 | Y | Y | Y | N | NA | Y | N | U | Y | Y | 66.6 |
| Weitzel 2020 | Y | Y | N | Y | NA | Y | Y | U | Y | N | 66.6 |
| Yamayoshi 2020 | Y | Y | Y | N | NA | Y | N | U | Y | Y | 66.6 |

1. Was a consecutive or random sample of patients enrolled? 2. Was a case control design avoided? 3. Did the study avoid inappropriate exclusions? 4. Were the index test results interpreted without knowledge of the results of the reference standard? 5. If a threshold was used, was it pre-specified? 6. Is the reference standard likely to correctly classify the target condition? 7. Were the reference standard results interpreted without knowledge of the results of the index test? 8. Was there an appropriate interval between index test and reference standard? 9. Did all patients receive the same reference standard? 10. Were all patients included in the analysis? Y: Yes, N: No, U: Unclear, NA: Not applicable
